# Cost-Effectiveness of Total Hip and Knee Replacements for the Australian Population with Osteoarthritis: Discrete-Event Simulation Model

**DOI:** 10.1371/journal.pone.0025403

**Published:** 2011-09-23

**Authors:** Hideki Higashi, Jan J. Barendregt

**Affiliations:** School of Population Health, The University of Queensland, Herston, Queensland, Australia; Erasmus University Rotterdam, The Netherlands

## Abstract

**Background:**

Osteoarthritis constitutes a major musculoskeletal burden for the aged Australians. Hip and knee replacement surgeries are effective interventions once all conservative therapies to manage the symptoms have been exhausted. This study aims to evaluate the cost-effectiveness of hip and knee replacements in Australia. To our best knowledge, the study is the first attempt to account for the dual nature of hip and knee osteoarthritis in modelling the severities of right and left joints separately.

**Methodology/Principal Findings:**

We developed a discrete-event simulation model that follows up the individuals with osteoarthritis over their lifetimes. The model defines separate attributes for right and left joints and accounts for several repeat replacements. The Australian population with osteoarthritis who were 40 years of age or older in 2003 were followed up until extinct. Intervention effects were modelled by means of disability-adjusted life-years (DALYs) averted. Both hip and knee replacements are highly cost effective (AUD 5,000 per DALY and AUD 12,000 per DALY respectively) under an AUD 50,000/DALY threshold level. The exclusion of cost offsets, and inclusion of future unrelated health care costs in extended years of life, did not change the findings that the interventions are cost-effective (AUD 17,000 per DALY and AUD 26,000 per DALY respectively). However, there was a substantial difference between hip and knee replacements where surgeries administered for hips were more cost-effective than for knees.

**Conclusions/Significance:**

Both hip and knee replacements are cost-effective interventions to improve the quality of life of people with osteoarthritis. It was also shown that the dual nature of hip and knee OA should be taken into account to provide more accurate estimation on the cost-effectiveness of hip and knee replacements.

## Introduction

Musculoskeletal conditions constitute a major burden to the Australian population. Over 6.1 million people (of a population of 20 million) were estimated to suffer from a musculoskeletal condition in 2004 [1]. Musculoskeletal conditions are among the most frequently managed diseases by general practitioners accounting for 17% of consultations in 2003–2004 [2]. In 2002, musculoskeletal conditions were selected as the 7^th^ National Health Priority Area [3]. Amongst the various conditions, osteoarthritis (OA) is the most common type. According to the Australian Burden of Disease and Injury study 2003 [4], OA accounted for 34,578 disability-adjusted life-years (DALYs) and was the largest contributor to musculoskeletal disease burden. The health expenditure associated with OA was AUD 1.1 billion in 2000-2001, or 25% of all musculoskeletal conditions that was the third largest component of the total health expenditure accounting for 9.4% [3]. One of the characteristics of OA is that the prevalence is higher amongst lower socio-economic quintiles, but not necessarily among the Indigenous population [5].

Currently there are limited measures to prevent OA, and there is no absolute cure [3]. However, various non-surgical and surgical procedures have become available to manage the symptoms associated with OA and improve physical mobility and quality of life of the affected population. Guidelines for non-surgical therapies and surgical referrals have been developed for general practitioners in Australia [2], [6]. Whilst several options are available for surgical interventions, joint arthroplasty for hips and knees have been shown to be particularly efficacious to improve the quality of life of people with OA [7]. Studies from Australia and other countries have demonstrated hip and knee replacements to be a cost-effective option [8], [9], [10], [11]. However, we are not aware of an analysis which accounted for the fact that people have two hips and two knees, and that within each pair joints are likely to have different severities of OA. Evaluations that take only a single joint into account may overestimate the benefit of surgical intervention because a sizable proportion of patients have OA in both joints, and pain relief will therefore be limited.

The aim of this study is to evaluate the cost-effectiveness of total hip and knee replacements for OA patients in Australia by accounting for two joints of the individuals. It was conducted as part of a large project which compares the cost-effectiveness of over 150 preventive and curative health interventions in Australia [12]. Therefore, this study complies with the economic protocol of the project in order to enable comparisons with all other interventions. This included the use of disability-adjusted life year (DALY) as the health outcome measure, which had a major implication on the methods to model the intervention effects.

## Methods

The economic protocol of the parent project of this study guided the choice of methods for this study. The following sections provide the details of methods employed for the cost-effectiveness analysis. Further details are provided in Text S1 that is available online.

### Perspective

A health system perspective was employed for this study. Although guidelines for economic evaluations often advocate a societal perspective, the broadest among various alternatives [13], the most appropriate perspective varies with each study depending on the target audiences and decision-making contexts. The parent project of this study aimed to assist policy makers and health services managers in making practical decisions about what services to provide within the available resources in the health sector. Given the aim, a health system perspective was deemed most appropriate for the project and was adopted in the economic protocol to which this study mostly adheres.

### Discrete-event simulation model

The analysis employed a discrete-event simulation (DES) model. This type of model has over 50 years of history as a major tool for operational research [14]. Originally developed in the steel industry [15], DES models expanded over various sectors particularly in manufacturing, industry and service sectors [16]. Although the application to the health sector has been less extensive [14], experts lists healthcare as one of growing priority areas for DES model application [17]. This is reflected in the annual number of publications using DES in the healthcare domain which has nearly doubled since 2004 [18]. Application in the healthcare setting has been typically in modeling situations where populations of patients interact with healthcare delivery system, (e.g. elective surgery, operation room management, transplantable organ-allocation, patient-flow forecasting), interaction of individuals (e.g. infectious diseases), resource planning, and economic evaluation [19], [20], [21].

In economic evaluations, DES model has the flexibility to accommodate a richer structure without making it unmanageable in size [20]. Our study models OA stage progression, decision for joint replacement, durability and time of repetitive revisions for each joint separately, which requires a large number of attributes and events that likely exceeds the manageable size of a Markov model. Further, the time to event (e.g. decision for replacement, revision) depends on the time the joints have spent in the previous attribute. Such “memories” can be attached to the individuals in a DES model, which is difficult to achieve with a cohort Markov approach [20]. In a DES model, individual life histories are created by drawing randomly from distributions that describe the time to the occurrence of particular events. The individuals from the study population would move from one attribute to another, driven by events, by means of time to progression of OA severity, time to decision for surgery, probability of surgical success and death, survival time of implants to revisions, and time to death. We accounted for the right and left joints for each individual, with in the baseline analysis the assumption that the attributes of hip or knee joints are independent of each other (this assumption was examined in a sensitivity analysis (see Text S1 Section 3.3).

**Figure 1:**
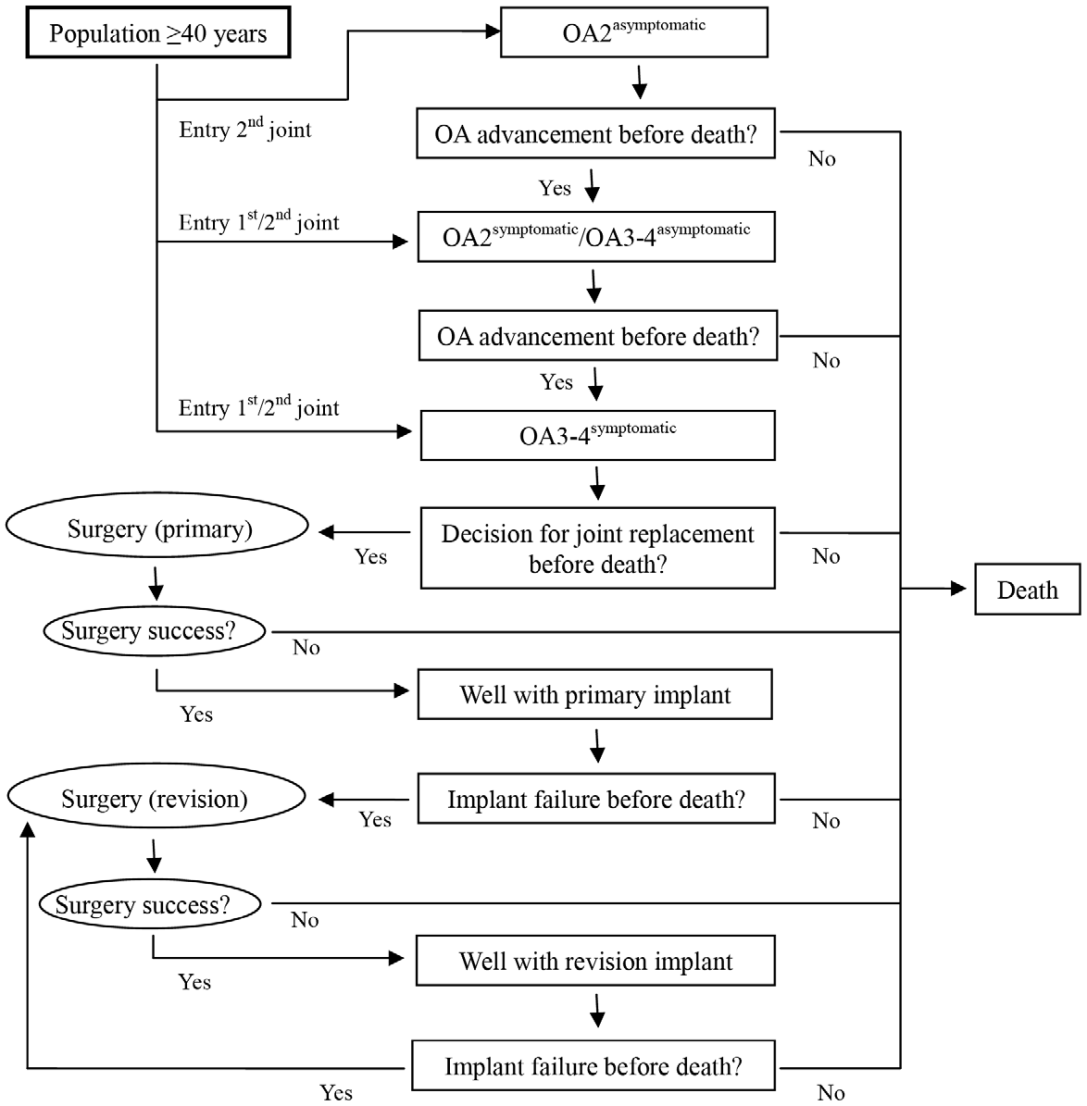
Event pathway of people with osteoarthritis in the discrete-event simulation model.

Figure 1 provides the schematic depiction of the model. An individual aged 40 or over in 2003 enters the model if the person has at least one joint with moderate OA (grade 2 with symptoms or grade 3–4 without symptoms) or worse. The other joint may or may not have OA, but both joints are at risk for progression of OA severity (or incidence) for some time. Following the assumption set for the Burden of Disease Study [4], the background mortality rate for these people was assumed to be higher with a relative risk of 1.1 compared to the rest of population without OA. The person with OA will receive non-surgical therapies, such as simple analgesia, non-steroidal anti-inflammatory drugs, land-based exercise and others, until the OA reaches a severity level that becomes too difficult to manage. Once the decision for a joint replacement is made, the person will receive the surgery which may either be successful (the DW of the joint will improve by the estimated effect size, either with complications or without) or unsuccessful (surgical death). After a successful replacement of a joint, the implant will function for a certain time until an event prompts a revision (e.g. dislocation, loosening etc.). The process of surgery and implant failure will repeat for each OA affected joint for life.

**Figure 2:**
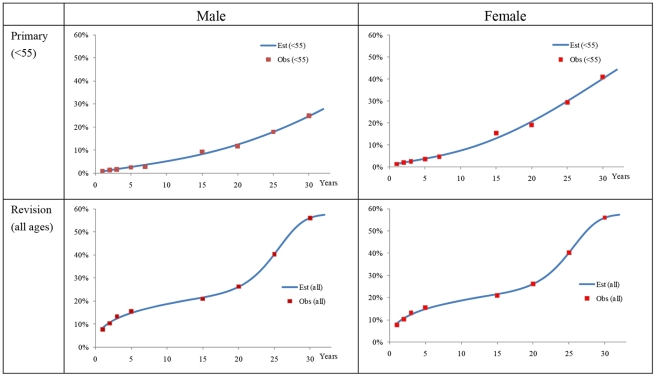
Cumulative distribution curve of time to revision with observed values (hip implants).

In modelling such events for each individual, we have drawn parameters from various sources. The information on the progression of OA severities was obtained from the Bristol OA 500 study [22] which followed-up patients with OA for eight years. The time to decision of surgery was modelled from the annual surgical rate among severe OA patients, which was calculated as the proportion of surgeries reported in the National Joint Replacement Registry [23] for 2003 and the number of OA patients at grade 3–4 (symptomatic) from the Burden of Disease study. The time to revision of implants was modelled by distinguishing short- and long-term causes. The information on the time to failure of implants due to short-term causes was obtained from the revision rates over seven years reported in the National Joint Replacement Registry [24]. The information on long-term causes was obtained from follow-up studies in international literature. We assumed separate Weibull distributions for short- and long-term causes for implant failures. The probability density curves for implant failures from the two Weibull distributions were then combined to a mixture distribution, normalized (i.e. the surface under the mixture distributions equals unity) and adjusted to fit the observed values by means of the Solver function in MS-Excel. Figure 2 provides an example of the combined distribution curve of time to revision of hip implants (see Text S1 Section 1.9 for further details of methods and outputs).

In simulating the progression of OA severity, time to decision of surgery and time to implant failure for each individual, we converted each estimated parameter to a continuous survival curve from which random draws determined the time to the next events. Other events, such as death from surgery, were determined by assessing the outcome of a Bernoulli trial based on the probabilities of such events obtained from literature (see Text S1 Section 1.6). The model was implemented in MS-Excel, using the Ersatz add-in [25] to perform the simulation. Table 1 provides the sources of information from which the input parameters were obtained.

**Table 1:** Data sources.

Parameters	Sources of information
***Population and demographic***	
Population	Burden of Disease 2003 [4]
Mortality rate	Burden of Disease 2003
Prevalent years lived with disability	Burden of Disease 2003
***OA***	
Prevalence (all)	National health survey 2001 [1]
Prevalence (grade 2 symptomatic+)	Burden of Disease 2003
Proportion of number of people in each grade	Burden of Disease 2003, literature [26], [27]
Mortality relative risk (OA)	Burden of Disease 2003
Progression of OA severity	Literature[22], Burden of Disease 2003
Proportion of bilateral OA	Literature [28], [29], [30], [31], [32], [33], [34], [35], [36]
	
DW	Burden of Disease 2003
***Intervention (hip and knee replacement)***	
Proportion of OA as primary diagnosis	Joint replacement registry 2007 [37]
Number of operations	Joint replacement registry 2004 [23]
Surgical death rate	CIHI 2007 [38], Joint replacement registry 2007
Revision rate (short term)	Joint replacement registry 2008 [24]
Revision rate (long term)	Literature [39], [40], [41], [42], [43], [44], [45]
Effect	Literature [7], [46], [47], [48], [49], [50], [51], [52], [53], [54], [55], [56], [57], [58]
***Cost***	
Hip and knee replacement surgery	Australian hospital statistics (2003-04), National hospital cost data collection (2003–2004) [59], [60]
Health expenditure for OA and all other health conditions	Disease costing and impact study (2000-01) [61]
Patient's out of pocket payment	Literature [62]
Patient's time cost	Average weekly earnings [63], Labour Force Statistics 2003 [64]
Price deflator	Health expenditure in Australia (2003-04) [65]

OA: osteoarthritis; CIHI: Canadian Institute for Health Information.

### Study Population

The study population comprised of Australians with OA who were 40 years of age or older in 2003 as was estimated in the Australian Burden of Disease and Injury study. In the Burden of Disease study, OA was divided into four grades with different disability weights (DWs) assigned (see Table 2 and Text S1 Section 1.8) [4], [66]. We limited the inclusion to males and females who had at least one hip or knee with grade 2 symptomatic OA or higher. The number of individuals for each sex/age-group was obtained from the Burden of Disease study. The study did not distinguish people with hip and knee OA. We therefore assumed that the proportion of hip and knee OA was the same as the age-group specific proportions of hip and knee replacement surgeries conducted in Australia in 2003 [23]. 68,908 individuals (30,347 males and 38,561 females) with hip OA and 100,657 individuals (42,930 males and 57,727 females) with knee OA entered the analysis and were followed-up until extinct.

**Table 2:** Case definition and sequelae.

OA sequelae	Definition
Grade 2 (radiological)	Definite osteophytes in hip or knee
Grade 2 (symptomatic)	Grade 2 and pain for at least 1 month in last 12
Grade 3-4 (asymptomatic)	Osteophytes and joint space narrowing in hip or knee,deformity also present for Grade 4
Grade 3-4 (symptomatic)	Grade 3+ and pain for at least 1 month in last 12

OA: osteoarthritis.

Source: The burden of disease and injury in Australia 2003 [4].

### Interventions

The interventions are total replacement of hips and knees. Whilst alternative methods are available for primary surgeries (i.e. hip resurfacing, uni-compartmental knee arthroplasty etc.), only primary conventional total hip replacements and primary total knee replacements, as defined in the National Joint Replacement Registry [23], including their subsequent revisions, were considered in this analysis as they constitute the majority of surgeries in Australia (91% of OA primary hip, 86% of OA primary knee) [24], and the evidence on efficacy of other types of implants has yet to be well established. All patients were assumed to have received a series of non-surgical treatments until these become insufficient prior to the decision to undergo surgery. The comparator for both hip and knee replacements is ‘doing nothing’ (continued non-surgical therapies without joint replacements).

OA is a chronic non-fatal disease which significantly affects the well-being of patients. Surgical intervention primarily aims to improve the quality of life of people with OA. The efficacies of hip and knee replacement surgeries have been evaluated by various instruments in the literature. Such instruments can range from generic (e.g. SF-36: Medical Outcomes Study Short-Form 36; HAQ: Health Assessment Questionnaire) [67], [68], arthritic-specific (e.g. WOMAC: Western Ontario and McMaster University Osteoarthritis Index) [69], or utility (e.g. EQ-5D: EuroQol 5-dimensions [70]). As mentioned, we used the DALY disability weight (DW) from the Burden of Disease study to quantify the impact of OA on the quality-of-life faced by OA patients. However, we were not able to identify any literature utilizing this instrument to measure the effect of hip and knee replacement. Therefore, we estimated the effect size on the DW from literature utilizing other instruments in the following manner:
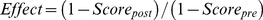
(1)

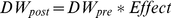
(2)where *Effect* denotes the effect size of an intervention, *Score_post_* the single index of post-surgery scores from other instruments, *Score_pre_* the single index of pre-surgery score of other instruments, *DW_post_* the DW at post-surgery, and *DW_pre_* the DW at pre-surgery. For instruments that do not use scores that fall between 0 and 1, we adjusted the scores to fall within this range (e.g. HAQ uses scores between 0 and 3, so we divided the scores by 3). The underlying assumptions for this novel approach were two. First, although the scores used in other instruments are fundamentally different and are not comparable to each other, the ratios between pre- and post-intervention scores in each instrument reflect the same relative health improvements from the intervention. Second, the effect sizes calculated as per the above equation are comparable between instruments and serve as proxies for the effects on the DWs. In order to test for the plausibility of this technique, a sensitivity analysis was performed to compare the cost-effectiveness results between DALY and EQ-5D as health outcome measures for hip replacements where both results proved fairly comparable (see Text S1 Section 3.3).

We used different approaches to calculate *Score_pre_* and *Score_post_* for hip and knee replacements depending on the available data. To calculate the scores for hip replacements, we used the regression model from Briggs et al. [46] which they employed to estimate the pre- and post-surgical quality of life scores of hip replacements using EQ-5D. We assumed normal distributions to each regression coefficient and calculated the pre- and post-surgical quality of life scores, which were then assigned to Expressions 1 and 2 to extrapolate the post-surgical DW.

On the other hand, we were not able to identify an appropriate source for knee replacement providing a regression model like this. Therefore, we used the literature included in a systematic review [7] reporting the pre-and post-surgical scores of knee replacements in EQ-5D, HAQ, and SF-36. We performed a non-parametric bootstrap on Expression 1 with 5,000 iterations by assigning the scores reported in 13 primary studies with 16 indexes (i.e. one EQ-5D, one HAQ, and fourteen SF-36 indexes) to derive the mean effect sizes and confidence intervals [47], [48], [49], [50], [51], [52], [53], [54], [55], [56], [57], [58], [71]. In order to derive a single score from studies using SF-36, we referred to the transfer to utility (TTU) technique, a tool developed by Segal et al. [8] to convert the multiple sub-scales of SF-36 to a single utility score for OA and subsequently applied to stroke [72]. In recognition of the critique raised by Viney et al. [73] regarding the TTU due to the fundamental differences between the concepts underlying health-related quality of life-scores and utilities, our aim was to estimate the effect size of the intervention under the assumptions set out above rather than to estimate the utility score itself. In light of the purpose of this study to compare the cost-effectiveness of disparate interventions, and in the absence of evidence on the effect size of hip and knee replacements on DWs, we regarded the TTU technique as an acceptable tool. Table 3 shows the estimated effect sizes on DWs (refer to Text S1 Section 1.8 for further details).

**Table 3:** Effect size of hip and knee replacements on disability-weights.

Joint	Type	Sex	Mean	SD	LCI 95%	HCI 95%
Hip	Primary	Male	0.3358	0.0454	0.2548	0.4319
		Female	0.3479	0.0376	0.2793	0.4260
	Revision	Male	0.5339	0.0830	0.3883	0.7115
		Female	0.5527	0.0709	0.4256	0.7018
Knee	Primary	Male	0.5202	0.0697	0.3888	0.6606
		Female	0.5205	0.0687	0.3891	0.6580
	Revision	Male	0.6610	0.0492	0.5642	0.7573
		Female	0.6698	0.0474	0.5772	0.7621

SD: standard deviation; LCI: lower confidence interval limit; HCI: higher confidence interval limit.

The health gains from the interventions were expressed as DALYs averted which were calculated using the following equation:
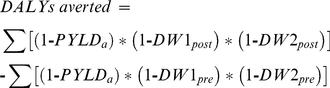
(3)where *PYLD_a_* denotes the prevalent years lived with disabilities (excluding those due to OA) of a person at age *a* since the time of primary joint replacement (for the intervention arm) or the time since the person was eligible for primary replacement should the person were to receive it (for the comparator arm), and *DW1_post/pre_* and *DW2_post/pre_* the disability experienced by the first and second joints for the person with and without joint replacements.

### Intervention Cost and Cost Offset

 Given the health system perspective employed for this study, all costs that fall on the health sector with the interventions were included in the analysis, both in the government and private sectors. The private sector costs included those born by the patients such as co-payment, travel costs and time costs. Health sector costs that can be saved due to the interventions are also included in the analysis.

Costs for surgeries were derived from the average cost per diagnosis-related group in 2003 [59], [60] and the disease costing and impact study 2000-2001 [61]. We distinguished the surgical costs between primary replacements and revisions, and with and without complications. The probability of having complications for primary replacements and revisions were extrapolated from the proportion of complications in 2003, which were estimated from the reports of National Joint Replacement Registry and cost per diagnosis-related group. A random value from the Bernoulli trial determined the presence or absence of complications for the individuals in the simulation (see Text S1 Section 2.1).

 Patient's out of pocket costs related to surgery were obtained from March et al. [62] assuming that the personal spending which accrue three months pre- and post surgery is part of the intervention costs. Patient's time costs were defined as those directly involved in receiving surgery-related services including travelling, waiting, pre-surgical visits, operation and recuperation. Unit costs associated with patient's time were obtained from the average weekly earnings in 2003 [63]. Future costs which are to be offset as the result of interventions were calculated from the annual OA expenditure obtained from disease costing and impact study 2000-2001 [61]. Unrelated health care costs, which would accumulate in the future due to extended life years of OA patients after joint replacements, were also obtained from the same source. Further details of the estimation of different costs are provided in Text S1 Section 2. The full data sources and estimated intervention costs are summarized in Tables 1 and 4. The exchange rate for Australian dollar (AUD) in 2003 was 1 AUD = 0. 67 USD (07/01/2003) [74]. The costs obtained from different years were all adjusted to the AUD 2003 value by means of price deflator [65].

**Table 4:** Intervention cost (per surgery).

Cost item	Cost per surgery (AUD)
***Government cost***	
Hip replacement surgery (primary–Cscc)	13,648
Hip replacement surgery (primary+Cscc & revision–Cscc)	16,744
Hip replacement surgery (revision+Cscc)	30,648
Knee replacement surgery (primary–Cscc)	13,640
Knee replacement surgery (primary+Cscc & revision–Cscc)	19,620
Knee replacement surgery (revision+Cscc)	35,912
Other costs related to surgery (non-admitted visits etc.)	2,254
***Patient out of pocket cost***	
Out of pocket cost pre- and post-surgery (hip)	839
Out of pocket cost pre- and post-surgery (knee)	1,019
***Time cost***	
Pre-surgical visits (hip)	168
Surgery & recuperation (hip, male, primary–Cscc)	2,227
Surgery & recuperation (hip, male, primary+Cscc & revision–Cscc)	3,781
Surgery & recuperation (hip, male, revision+Cscc)	5,629
Surgery & recuperation (hip, female, primary–Cscc)	1,576
Surgery & recuperation (hip, female, primary+Cscc & revision–Cscc)	2,677
Surgery & recuperation (hip, female, revision+Cscc)	3,985
Pre-surgical visits (knee)	171
Surgery & recuperation (knee, male, primary–Cscc)	2,096
Surgery & recuperation (knee, male, primary+Cscc & revision–Cscc)	4,197
Surgery & recuperation (knee, male, revision+Cscc)	6,246
Surgery & recuperation (knee, female, primary–Cscc)	1,484
Surgery & recuperation (knee, female, primary+Cscc & revision–Cscc)	2,970
Surgery & recuperation (knee, female, revision+Cscc)	4,422

Cscc: catastrophic or severe complications and comorbidities.

### Accounting for Uncertainties

Uncertainty distributions were provided for input parameters where appropriate in order to account for sampling uncertainties. The model underwent Monte Carlo simulation (often also known as probabilistic sensitivity analysis) by re-sampling the values of parameters 2,000 times from the given distributions. The distributions provided for each parameter are shown in Table 5.

**Table 5:** Distributions assumed for each parameter.

Parameters	Distributions
Time to primary replacement of hip and knee joints*	Empirical
Time to death*	Empirical
Time to revision of hip and knee implants*	Weibull†
Intervention effect (regression coefficients for hip replacement)	Normal‡
Intervention effect (knee replacement)	Beta‡
Intervention cost (hip and knee surgeries)	Gamma§
Patient's out of pocket payment pre/post-surgeries	Gamma§, Triangular**
Patient's time cost for surgeries	Gamma§, Triangular**
Average length of stay for hip and knee surgeries and recuperations	Gamma§

*These parameters accounted for the first-order uncertainties (individual level), and others the second-order uncertainties (population level).

† Time to failure due to short-run and long-run causes were distinguished. We assumed separate Weibull distributions for each cause, and modelled the time to revision as the normalized sum of these two. The Weibull parameters are provided in Text S1 Section 1.9.

‡ The values are provided in Text S1 Section 1.8.

§ The parameters of Gamma distributions consist of Alpha  =  unit cost, Beta  =  1.

**Triangular distribution was used to model the uncertainty of unit costs (±20%), and Gamma distribution for the time spent in hospital.

The model was implemented in Microsoft Excel 2007, with the Ersatz add-in [25] for uncertainty analysis. We conducted the simulation under different scenarios based on the inclusion or exclusion of cost offset and patient's time cost. Future costs and health gains were discounted at an annual rate of 3% to account for time preference. The internal consistency of model was tested by comparing the proportions of total joint replacements occurring in each sex/age-group for a given year between the joint registry [23] and our simulation.

## Results

**Figure 3:**
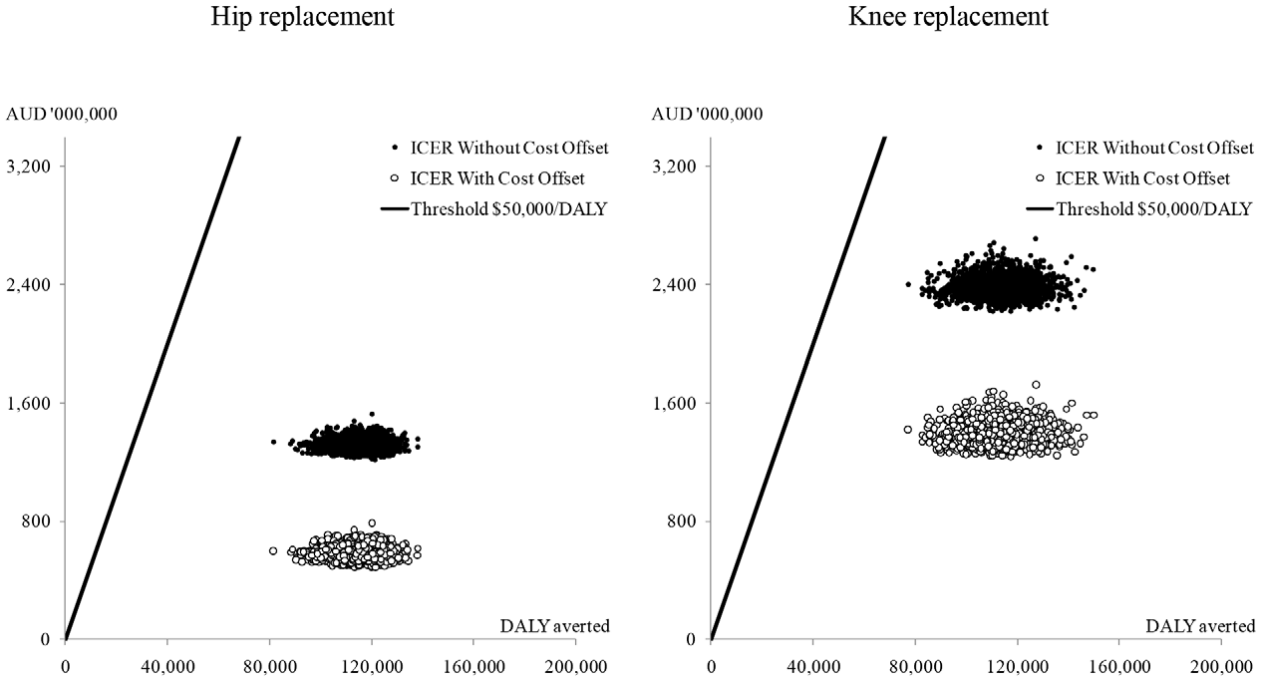
Cost-effectiveness of hip and knee replacements illustrated on a cost-effectiveness plane with AUD 50,000 per DALY threshold line (without time cost). The graphs represent population total rather than per patient outcomes (hip replacement: 68,908 individuals; knee replaceement: 100,657 individuals).

Among those who have undergone successful hip and knee replacement surgeries at least once, 49% of males and 42% of females with hip OA had their second joints replaced along their course of life. The proportions of bilateral replacements for people with knee OA were 57% and 52% for males and females respectively. The test for internal consistency provided reasonably comparable outputs between the joint registry and our simulation (see Text S1 Section 3.4). Tables 6, 7, and 8 provide the costs, health gains, and incremental cost-effectiveness ratios (ICERs) and 95% uncertainty intervals (UIs) (sex-specific results provided in Text S1 Section 3.1). The results are reported at the population-level due to the aim of this study to inform policy makers at the national-level. While the protocol of the parent project of this study excluded the unrelated health care costs that would accumulate in the future due to prolonged life-years of patients [12], we report the results with the inclusion of such costs as an additional scenario (Table 9). Both hip and knee replacements were cost-effective compared to the pre-defined threshold level of AUD 50,000 per DALY averted by the overarching project of this study [12]. Although the ICERs become less favourable if we excluded the cost offset, or included the future unrelated health care costs, they are consistently below the threshold level. The scatter plots of hip and knee replacements are provided in Figure 3.

**Table 6:** Health gains.

	Hip (DALY averted)		Knee (DALY averted)	
	Mean	95%UI	Mean	95%UI
DALY averted	115,000	98,800 – 129,000	113,000	93,200 – 133,000
DALY averted (per person)*	1.7 per person		1.1 per person	

DALY: disability-adjusted life-years; UI: uncertainty interval.

NB: The values are discounted by 3%, and rounded to the three digits of significance.

* Mean value divided by the number of people (68,908 for hip, 100,657 for knee).

**Table 7:** Costs under different scenarios.

Scenario	Hip (AUD Millions)		Knee (AUD Millions)	
	Mean	95%UI	Mean	95%UI
*With cost offset*				
Without time cost	420	400 – 440	1,100	1,100 – 1,200
With time costs	580	520 – 670	1,400	1,300 – 1,500
*Without cost offset*				
Without time cost	1,200	1,100 – 1,200	2,100	2,100 – 2,200
With time costs	1,300	1,300 – 1,400	2,400	2,300 – 2,500
*Cost per person* *				
With cost offset (without time costs)		6,100 per person	11,000 per person	
Without cost offset (without time cost)		17,000 per person	21,000 per person	

AUD: Australian Dollar; UI: uncertainty interval.

NB: The values are discounted by 3%, and rounded to the two digits of significance.

*Mean value without time cost (unit: AUD) divided by the number of people in the model.

**Table 8:** Incremental cost-effectiveness ratio.

Scenario	Hip (AUD per DALY averted)		Knee (AUD per DALY averted)	
	Mean	95%UI	Mean	95%UI
*With cost offset*				
Without time cost	3,600	3,200 – 4,200	10,000	8,400 – 12,000
With time costs	5,000	4,200 – 6,200	12,000	10,000 – 15,000
*Without cost offset*				
Without time cost	10,000	9,000 – 12,000	19,000	16,000 – 23,000
With time costs	12,000	10,000 – 13,000	21,000	18,000 – 26,000

AUD: Australian Dollar; DALY: disability-adjusted life-years; UI: uncertainty interval.

NB: The values are discounted by 3%, and rounded to the two digits of significance.

**Table 9:** Incremental cost-effectiveness ratio including future unrelated health care costs.

Scenario	Hip (AUD per DALY averted)		Knee (AUD per DALY averted)	
	Without time cost	With time cost	Without time cost	With time cost
*With cost offset*	7,100	8,600	15,000	17,000
*Without cost offset*	13,000	15,000	24,000	26,000

AUD: Australian Dollar; DALY: disability-adjusted life-years.

NB: The values are discounted by 3%, and rounded to the two digits of significance.

## Discussion

This study has found favourable cost-effectiveness for hip and knee replacement in Australia. Given the sizable burden of OA in Australia, the interventions contribute substantially to the improvement of people's quality of life at reasonable costs. However, there were substantial differences in the cost-effectiveness between hip and knee replacements. Hip replacements were substantially more cost-effective than knee replacements. The ICER for knee replacements without cost offsets was AUD 21,000, or AUD 26,000 including unrelated health care costs, per DALY averted with time costs, and was about half that for hip replacements (AUD 12,000, or AUD 15,000 including unrelated health care costs, per DALY). The difference became slightly more prominent if we included the cost offsets in the analysis (ICER for knee: AUD 12,000, or AUD 17,000 including unrelated health care costs, per DALY with time costs; ICER for hip: AUD 5,000, or AUD 8,600 including unrelated health care costs, per DALY). There are a number of reasons for the more favourable results of hip replacements. First, the post-surgery health outcomes for hip replacements consistently surpass that for knee replacements in the literature [7]. This was reflected in our result where the cumulative health benefits was similar for hip and knee replacements despite the smaller number of people included in the hip replacement analysis (see Table 6). Second, more revisions were required for knee replacements (see Text S1 Section 1.10). Since a hip replacement provides better health outcome at lower costs compared to a knee replacement, it is more cost-effective. Although females have slightly more favourable ICERs for both hip and knee replacements, the UIs largely overlap with those of males. On the other hand, the ICERs can substantially vary between age-groups. Age-group specific results are provided in Text S1 Section 3.2 which shows less favourable ICERs for elderly patients, yet mostly within the threshold level.

An earlier study in Australia by Segal et al. [8] also reported favourable cost-effectiveness for joint replacements (AUD 4,535 – 6,953 per QALY for hip replacement and AUD 7,671 – 11,671 for knee replacement) although the findings are noticeably more favourable than ours (AUD 9,000 – 12,000 per DALY for hip replacement and AUD 16,000 – 23,000 per DALY for knee replacement, without cost offset, time cost, and unrelated health care cost) and not directly comparable due to methodological differences. Studies from other countries suggest the cost-effectiveness of hip replacement ranges between cost-saving and AUD 10,900 per QALY, and between AUD 9,000 and 24,400 per QALY for knee replacement [9], [10], [11]. In order to make the results from our study more comparable to the other ones, we conducted a sensitivity analysis by restricting one joint of all individuals to be free from OA for life (unilateral OA for both hips and knees), and by replacing the health outcome measure from DALY to QALY (EQ-5D) for hip replacement (this was difficult for knee replacement since the employed effect size was a pooled product from multiple studies using different instruments). The results from the analysis where one joint was always free from OA were AUD 6,900 per DALY and AUD 11,000 per DALY for hip and knee replacements, respectively (Text S1 Section 3.3). Further, replacing the health outcome measure for hip replacement to QALY and restrict one hip to be free from OA provided a cost-effectiveness ratio of AUD 6,500 per QALY (Text S1 Section 3.3). Interestingly all these results fell within the range of the ones from Segal et al., which support the hypothesis that accounting for only one joint in the analysis yields too favourable cost-effectiveness ratios for hip and knee replacement surgeries.

 Our analysis made a number of assumptions and has some limitations which are worth noting. First the quantification of the intervention effects was problematic. Whilst the change in the DALY DW plays a key role in measuring the intervention effects, we were not able to identify studies utilizing this instrument to measure the effects of hip and knee replacements. Therefore we extrapolated the effects from other instruments with assumptions which potentially could have under- or over-estimated the true DW post-interventions. However, the extrapolations were in line with the findings from a systematic review that the post-replacement indexes are consistently better than pre-surgery, and hip replacements have consistently better health outcomes than knee replacements. Further, as mentioned above, the sensitivity analysis comparing the results between DALY and EQ-5D as health outcome measures suggested the plausibility of these assumptions.

Another limitation was that the durability of hip and knee implants were modelled from historical data, which may be under estimating the current survivorship of implants given the technological advancement over decades. This issue has potentially resulted in a less favourable cost-effectiveness, since an improved durability of implants would reduce the number of revisions. Further, the revision rates of hip implants obtained from the National Joint Replacement Registry included replacement cases due to fractured neck of femur which are known to have a shorter lifespan than from other causes and were not included in our study population. This may have potentially caused an under-estimation of the true survivorship of implants. However, the proportion of replacements due to fractures is small (2.8% between 1999 and 2004) [75], and so is not likely to have affected the estimation substantially.

The independence of OA progression and time to revision of implants assumed for the right and left joints warrants due attention. Whilst some correlation may exist between the two joints, the actual degrees are unknown. Therefore we performed a sensitivity analysis by assuming an extreme correlation between the two joints; i.e. ±0.99. The analysis did not provide significant deviations from our original results (see Text S1 Section 3.3), and so our findings are robust with respect to this assumption.

On the other hand, the study has its own strength. The nature of the intervention, which may or may not require repeated revisions at varying intervals for one or two joints, favoured the employment of a DES model. The model has the potential to account for variations at both (or either) individual levels (first order) and population levels (second order). This is one of the advantages of this study which would reflect the variations at the population level more accurately. Another strength was that the model was able to account for two hips or knees for each individual with OA. Modelling two joints separately for a person would have been difficult with other approaches. As discussed above, modelling just one joint overestimates health benefits since a sizable proportion of OA patients suffer from bilateral problems and hence replacement of one joint will leave the problem on the other joint. In this regard, a further study that account for OA problems in multiple sites of a patient (i.e. both hip and knee joints) may be warranted as more epidemiological data on OA become available.

In conclusion, the findings suggest that both hip and knee replacements are highly cost-effective with ICERs significantly lower than the AUD 50,000 per DALY threshold level. The interventions substantially contribute to the improvement of quality of life of population suffering from OA. Despite the limitations of the study, the overall conclusions from the analysis are not likely to be affected. It was also shown that we should take the dual nature of hip and knee OA into account to provide more accurate estimation on the cost-effectiveness of hip and knee replacements.

## Supporting Information

Text S1 Details of methods, input data and additional results.
(PDF)
