# Supplementary material for: Cost-Effectiveness of Total Hip and Knee Replacements for the Australian Population with Osteoarthritis: Discrete-Event Simulation Model
Source: PLoS One. 2011 Sep 23;6(9):e25403. doi: 10.1371/journal.pone.0025403 (PMC3179521; doi:10.1371/journal.pone.0025403)
Supplement: Text S1 — Details of methods, input data and additional results. (PDF) [file pone.0025403.s001.pdf]

# APPENDIX

Cost-effectiveness of total hip and knee replacements
for the Australian population with osteoarthritis:
discrete-event simulation model

Hideki Higashi, Jan J. Barendregt

## *Contents*


**1. Epidemiological modelling ........ 3**

1.1. Model outline ........ 3
1.2. Number of people with hip and knee OA in 2003 ........ 3
1.3. Proportion of bilateral hip and knee OA ........ 6
1.4. Time to primary joint replacement ........ 6
1.5. Progression of OA to a higher severity ........ 7
1.6. Probability of dying from hip or knee replacement surgeries ........ 8
1.7. Probability of dying from non-surgical causes among people with OA ........ 8
1.8. Health gains ........ 9
1.9. Time to revision of hip and knee implants ........ 12
1.10. Survival curve of implants (primary joint replacement) ........ 18

**2. Costs ........ 19**

2.1. Costs of hip and knee replacement surgeries ........ 19
2.2. Average length of stay ........ 20
2.3. Surgery-related costs ........ 21
2.4. Patient’s out-of-pocket costs ........ 21
2.5. Patient’s time costs for surgery and recuperation ........ 22
2.6. Patient’s time costs for pre-surgery medical visits ........ 22
2.7. Cost offsets (non-surgical therapies for OA) ........ 23

**3. Additional results ........ 25**

3.1. Sex-specific results ........ 25
3.2. Age-group specific results ........ 25
3.3. Results from sensitivity analyses ........ 26
3.4. Testing for internal consistency ........ 27

**Reference ........ 28**

# 1. Epidemiological modelling

## 1.1. Model outline

The cost-effectiveness analysis of hip and knee replacements employs a discrete-event simulation (DES) model that follows individuals with OA throughout their life courses. The schematic depiction of the model was provided in the Figure 1 of the main text, and hence is not reproduced here. Table A lists all patient attributes and events with the corresponding Sections of this Appendix where the details of the relevant parameters are provided.

Table A: Attributes and events that forme the model

| | Parameter description | Section |
|---|---|---|
| *Patient attributes* | | |
| OA grade 2 (asymptomatic) | OA associated with minor DW | 1.8 |
| OA grade 2 (symptomatic) | OA associated with moderate DW | 1.8 |
| OA grade 3-4 (asymptomatic) | OA associated with moderate DW | 1.8 |
| OA grade 3-4 (symptomatic) | OA associated with severe DW | 1.8 |
| Well with primary implant | Reduced DW associated with primary implants | 1.8 |
| Well with revision implant | Increased DW associated with revised implants | 1.8 |
| Death | Includes both deaths from surgery and all other causes | 1.6, 1.7 |
| *Events* | | |
| Entry into the model | All individuals aged 40 and older, with either unilateral or bilateral OA on hips or knees in Australia, 2003 | 1.2, 1.3 |
| OA progression | Time to progression of OA severity from a grade to the next | 1.5 |
| Primary replacement | Time to primary replacement surgery of hips or knees | 1.4 |
| Primary implant revision | Time to revision of primary implants due to some failures | 1.9 |
| Repeated implant revision | Time to revision of revised implants due to some failures | 1.9 |
| Dying from surgery | Probability of dying from hip or knee replacements | 1.6 |
| Dying from other causes | Probability of dying from causes other than hip or knee replacements | 1.7 |

OA: osteoarthritis; DW: disability weight

For the purpose of simplifications, the abbreviations provided in Table B are used to describe the OA grades in all parameters appearing in this document.

Table B: Abbreviations for OA severity

<table>
<tr><th>OA grade</th><th colspan="5">Abbreviations</th></tr>
<tr><td>Less than Grade 2</td><td><2A</td><td></td><td></td><td></td><td></td></tr>
<tr><td>Grade 2 (asymptomatic)</td><td>2A</td><td rowspan="4">2A+</td><td></td><td></td><td></td></tr>
<tr><td>Grade 2 (symptomatic)</td><td>2S</td><td rowspan="3">2S+</td><td></td><td rowspan="2">2S3A</td></tr>
<tr><td>Grade 3-4 (asymptomatic)</td><td>3A</td><td rowspan="2">3A+</td></tr>
<tr><td>Grade 3-4 (symptomatic)</td><td>3S</td><td></td></tr>
</table>

## 1.2. Number of people with hip and knee OA in 2003

The study population was calculated from data available from the Australian Burden of Disease study 2003 [1]. However, the estimated prevalence of people with OA was not divided into hips or knees in the study. In order to derive the number of people with hip OA and knee OA separately with different severities, we first calculated the number of people with OA (hip and knee mixed) with different grades as follows:

$$OAnum_{age}^{2A} = OAprev_{age}^{2A+} \times (1 - OAprop_{age}^{3A+}) \times (1 - OAprop_{age}^{2S}) \times Pop_{age}^{2003}$$

$$OAnum_{age}^{2S} = OAprev_{age}^{2A+} \times (1 - OAprop_{age}^{3A+}) \times OAprop_{age}^{2S} \times Pop_{age}^{2003}$$

$$OAnum_{age}^{3A} = OAprev_{age}^{2A+} \times OAprop_{age}^{3A+} \times (1 - OAprop_{age}^{3S}) \times Pop_{age}^{2003}$$

$$OAnum_{age}^{3S} = OAprev_{age}^{2A+} \times OAprop_{age}^{3A+} \times OAprop_{age}^{3S} \times Pop_{age}^{2003}$$

where

$OAnum_{age}^{2A/2S/3A/3S}$ is the number of people with OA at each severity and each age-group in 2003;

$OAprev_{age}^{2A+}$ is the OA prevalence rate with grade 2A or higher at each age-group in 2003 (obtained from the Burden of Disease study 2003);

$OAprop_{age}^{3A+/2S/3S}$ is the proportion of people with OA of grade 3A+, and the proportion of for grades 2S and 3S at each age-group derived from Felson and Guccione et al.[2,3]; and

$Pop_{age}^{2003}$ is the age-group specific number of Australian population in 2003.

Second, we calculated the number of hip and knee replacement surgeries due to OA that were conducted in Australia in 2003 [4]:

$$Snum_{age}^{hipOA} = Snum_{age}^{hip} \times SpropOA_{age}^{hip}$$

$$Snum_{age}^{kneeOA} = Snum_{age}^{knee} \times SpropOA_{age}^{knee}$$

where

$Snum_{age}^{hipOA/kneeOA}$ is the number of primary replacement surgeries for hip or knee (total hip and knee, hip resurfacing, unicompartmental knee) in each age-group in 2003 due to OA;

$Snum_{age}^{hip/knee}$ is the number of primary replacement surgeries for hip or knee (total hip & knee, hip resurfacing, unicompartmental knee) in each age-group in 2003 from all causes; and

$SpropOA_{age}^{hip/knee}$ is the proportion of people with OA who underwent primary replacement surgeries for hip or knee (total hip & knee, hip resurfacing, unicompartmental knee) in each age-group in 2003.

Finally, we derived the number of people with hip and knee OA in 2003:

$$OAnum_{age}^{hip2A/2S/3A/3S} = OAnum_{age}^{2A/2S/3A/3S} \times \frac{Snum_{age}^{hipOA}}{Snum_{age}^{hipOA} + Snum_{age}^{kneeOA}}$$

$$OAnum_{age}^{knee2A/2S/3A/3S} = OAnum_{age}^{2A/2S/3A/3S} \times \frac{Snum_{age}^{kneeOA}}{Snum_{age}^{hipOA} + Snum_{age}^{kneeOA}}$$

where

$OAnum_{age}^{hip/knee,2A/2S/3A/3S}$ is the number of hip or knee OA at each grade in each age-group in 2003.

The underlying assumption for the derivation was that the proportion between numbers of hip OA and knee OA is equivalent to the proportion between numbers of hip replacement and knee replacement surgeries conducted for OA in Australia.

Tables C and D provide the estimated number of people with hip and knee OA for grade 2S+ on at

least one joint for each age-group in 2003. These people entered the model.

Table C: Number of hip OA in each grade for each age-group in 2003

| Age | Male (Total 30,347) | | | Female (Total 38,562) | | |
|---|---|---|---|---|---|---|
| | 2S | 3A | 3S | 2S | 3A | 3S |
| 40-44 | 201 | 889 | 355 | 90 | 321 | 128 |
| 45-49 | 325 | 1,436 | 573 | 199 | 712 | 284 |
| 50-54 | 460 | 2,032 | 811 | 330 | 1,181 | 471 |
| 55-59 | 385 | 1,701 | 679 | 344 | 1,228 | 490 |
| 60-64 | 428 | 1,888 | 754 | 483 | 1,728 | 690 |
| 65-69 | 448 | 1,977 | 789 | 641 | 2,292 | 915 |
| 70-74 | 518 | 2,287 | 913 | 828 | 2,962 | 1,182 |
| 75-79 | 491 | 2,717 | 1,085 | 1,061 | 3,634 | 1,451 |
| 80-84 | 358 | 1,982 | 791 | 1,000 | 3,427 | 1,368 |
| 85-89 | 237 | 1,313 | 524 | 930 | 3,187 | 1,272 |
| 90-94 | 87 | 481 | 192 | 474 | 1,622 | 648 |
| 95-99 | 21 | 118 | 47 | 143 | 488 | 195 |
| 100+ | 6 | 34 | 14 | 28 | 96 | 38 |
| Total | 3,965 | 18,855 | 7,527 | 6,551 | 22,878 | 9,132 |

Table D: Number of knee OA in each grade for each age-group in 2003

| Age | Male (Total 42,930) | | | Female (Total 57,728) | | |
|---|---|---|---|---|---|---|
| | 2S | 3A | 3S | 2S | 3A | 3S |
| 40-44 | 201 | 889 | 355 | 82 | 295 | 118 |
| 45-49 | 325 | 1,437 | 573 | 183 | 653 | 261 |
| 50-54 | 460 | 2,032 | 811 | 303 | 1,084 | 433 |
| 55-59 | 561 | 2,475 | 988 | 576 | 2,057 | 821 |
| 60-64 | 622 | 2,748 | 1,097 | 810 | 2,895 | 1,156 |
| 65-69 | 749 | 3,308 | 1,321 | 1,126 | 4,024 | 1,606 |
| 70-74 | 867 | 3,827 | 1,528 | 1,454 | 5,198 | 2,075 |
| 75-79 | 837 | 4,637 | 1,851 | 1,871 | 6,407 | 2,558 |
| 80-84 | 611 | 3,384 | 1,351 | 1,764 | 6,042 | 2,412 |
| 85-89 | 238 | 1,318 | 526 | 965 | 3,306 | 1,320 |
| 90-94 | 87 | 482 | 193 | 491 | 1,683 | 672 |
| 95-99 | 21 | 119 | 47 | 148 | 507 | 202 |
| 100+ | 6 | 34 | 14 | 29 | 100 | 40 |
| Total | 5,585 | 26,690 | 10,655 | 9,802 | 34,251 | 13,674 |

## 1.3. Proportion of bilateral hip and knee OA

Once the number of people with hip OA and knee OA were derived with different severities, further modelling was required to estimate the proportion of people with bilateral OA in 2003. This was necessary in order to account for the dual nature of OA and simulate the progression of OA and surgeries between two joints separately. The proportion of bilateral OA was estimated from literature findings by means of weighted average[5-13].

$$BIprop_{BB}^{hip/knee} = \frac{\sum(BIprop_{n}^{hip/knee} \times N_n)}{\sum N_n}$$

$$BIprop_{BA}^{hip/knee} = 1 - BIprop_{2S3A\_2S3A}^{hip/knee}$$

$$BIprop_{2S3A\_3S}^{hip/knee} = BIprop_{2S3A\_2S3A}^{hip/knee} \times \frac{OAnum_{all\ age}^{\frac{hip}{knee},2S3A}}{OAnum_{all\ age}^{hip/knee,2S+}}$$

$$BIprop_{3S\_3S}^{hip/knee} = BIprop_{2S3A\_2S3A}^{hip/knee} \times \frac{OAnum_{all\ age}^{\frac{hip}{knee},3S}}{OAnum_{all\ age}^{hip/knee,2S+}}$$

$$BIprop_{3S\_2A}^{hip/knee} = 1 - BIprop_{2S3A\_3S}^{hip/knee} - BIprop_{3S\_3S}^{hip/knee}$$

where

2S3A_2A/2S3A_2S3A /2S3A_3S/3S_2A/3S_3S is an OA state with one hip/knee at state 2S3A or 3S, and the other one at state 2A, 2S3A B or 3S;

$BIprop_{2S3A\_2S3A\ /2S3A\_2A/2S3A\_3S/3S\_2A/3S\_3S}^{hip/knee}$ is the proportion of bilateral OA for each OA state;

$BIprop_{n}^{hip/knee}$ is the proportion of bilateral OA from the n[th] literature; and

$N_n$ is the sample size of the study in the n[th] literature.

The estimated probabilities to have bilateral OA from the above equations are provided in Table E.

Table E: Probability of having bilateral OA

| | Male | | Female | |
|---|---|---|---|---|
| Other joint (no.2) / Worse joint (no.1) | 2S3A | 3S | 2S3A | 3S |
| Hip 2S3A | 28.3% | - | 28.3% | - |
| Hip 3S | 21.3% | 7.0% | 21.6% | 6.7% |
| Knee 2S3A | 61.3% | - | 61.3% | - |
| Knee 3S | 46.1% | 15.2% | 14.5% | 46.8% |

## 1.4. Time to primary joint replacement

A person with OA is eligible for hip or knee replacement surgery once the person reached the highest OA severity (3S) and non-surgical therapies are no longer capable in managing the symptoms. However, not all individuals will immediately receive replacements due to waiting time and personal preference. In order to model the time to primary replacement surgery, we first calculated the remission rates (joint replacement rates) for hip and knee OA in 2003 for each age-group based on the

information from the Australian burden of disease and injury study as follows [1]:

$$REMnum_{age}^{2A+} = REMrate_{age}^{2A+} \times OAprev_{age}^{2A+} \times Pop_{age}^{2003}$$

$$REMrate_{age}^{hip/knee2S+} = \left[REMnum_{age}^{2A+} \times \frac{Snum_{age}^{hip/kneeOA}}{Snum_{age}^{hipOA} + Snum_{age}^{kneeOA}}\right] \div OAnum_{age}^{hip/knee2S+}$$

$$REMrate_{age}^{hip/knee3S} = \left[REMnum_{age}^{2A+} \times \frac{Snum_{age}^{hip/kneeOA}}{Snum_{age}^{hipOA} + Snum_{age}^{kneeOA}}\right] \div OAnum_{age}^{hip/knee3S}$$

where

$REMnum_{age}^{2A+}$ is the number of OA remissions (hip or knee replacement) in each age-group in 2003;

$REMrate_{age}^{2A+}$ is the remission rate (hip or knee replacement) of hip or knee OA at grade 2A or higher in each age-group obtained from ABOD 2003; and

$REMrate_{age}^{hip/knee2S+,hip/knee3S}$ is the remission rate (hip or knee replacement) of hip or knee OA at grade 2S or higher, or grade 3S estimated for each age-group in 2003.

The derived age-specific remission rates now provide the probability of undergoing replacement surgeries at each age which were given an Empirical distribution to model the time to primary hip or knee replacement in the simulation along the life course of each patient.

## 1.5. Progression of OA to a higher severity

As mentioned above, only persons with the highest severity of OA (3S) are eligible for the replacement surgery. The severity of OA that were less at the initial stage of the simulation will gradually progress over time until the joint becomes eligible for surgery. In order to model the progression of OA, we utilised information from literature and applied to the following equations:

$$OA_{age}^{2A\ to\ 2S3A} = 1 - e^{-\left(-\frac{\ln[1-\{(Mild0-Mild8)+(None0-None8)\}\div Mild8]}{8years}\right)}$$

$$OA_{age}^{2S3A\ to\ 3S} = \left[\left(OAnum_{age}^{2S3A} + OAnum_{age}^{3S}\right) \times \left(1 - REMrate_{age}^{2S+}\right) \times REMrate_{age}^{2S+} - REMrate_{age}^{3S} \times \left(OAnum_{age}^{3S} - OAnum_{age}^{3S} \times REMrate_{age}^{3S}\right)\right] \div \left(OAnum_{age}^{2S3A} \times REMrate_{age}^{3S}\right)$$

where

$OA_{age}^{2A\ to\ 2S3A;\ 2S3A\ to\ 3S}$ is the probability of a person to progress from OA grade 2A to 2S3A or from grade 2S3A to 3S at each age;

$Mild0/8$ is the number of people with OA (assumed comparable to grade 2A) at the beginning of study or eight years after in Dieppe et al.[14];

$None0/8$ is the number of people with OA (assumed comparable to grade <2A) at the beginning of study or eight years after in Dieppe et al.[14]; and

$8years$ is the length of follow up of people with OA in Dieppe et al.[14]

$OA_{age}^{2S/3A\ to\ 3S}$ was set to satisfy $OAnum_{age}^{2S+} \times REMrate_{age}^{2S+} = OAnum_{age}^{3S} \times REMrate_{age}^{3S}$

## 1.6. Probability of dying from hip or knee replacement surgeries

Once an OA patient becomes eligible for surgery and selects to undergo replacement, the person receives the operation. However, hip and knee replacement surgeries are not without the risk for mortality. Therefore the probability of surgical death was extrapolated from data obtained from the Canadian joint replacement registry[15], which was adjusted to distinguish the risks between primary and revision surgeries (Table F).

Table F: Probability of dying from surgery

| Age | Hip primary | Hip revision | Knee primary | Knee revision |
|---|---|---|---|---|
| <75 | 0.00198 | 0.00368 | 0.00099 | 0.00160 |
| 75-84 | 0.00853 | 0.01581 | 0.00387 | 0.00626 |
| 85+ | 0.04174 | 0.07731 | 0.00736 | 0.01189 |

The model was designed so that a random draw from a value between 0 and 1 is compared with the probability. If the drawn value is greater than the probability, the surgery was deemed success (with or without complications), while a smaller value results in surgical death. The following provides the mathematical background in estimating these probabilities

$$Sdeath_{Prim}{}_{age}^{hip/knee} = \frac{Sdeath_{CA}{}_{age}^{hip/knee}}{PropPrim_{CA}{}_{age}^{hip/knee} + PropRev_{CA}{}_{age}^{hip/knee} \times \dfrac{DeathRev_{AU}^{hip/knee}}{DeathPrim_{AU}^{hip/knee}}}$$

$$Sdeath_{Rev}{}_{age}^{hip/knee} = Sdeath_{Prim}{}_{age}^{hip/knee} \times \frac{DeathRev_{AU}^{hip/knee}}{DeathPrim_{AU}^{hip/knee}}$$

where

$Sdeath_{CA}{}_{age}^{hip/knee}$ is the surgical death rate in Canada for hip or knee replacement (primary and revision mixed) at each age-group;

$Sdeath_{Prim/Rev}{}_{age}^{hip/knee}$ is the extrapolated surgical death rate in Australia for hip or knee, primary or revision replacements for each age-group;

$PropPrim/Rev_{CA}{}_{age}^{hip/knee}$ is the proportion of primary and revision surgeries conducted for hips or knees in each age-group in 2005-2006, Canada; and

$DeathPrim/Rev_{AU}^{hip/knee}$ is the death rate per person years following hip or knee replacements for primary or revision surgeries between 1999 and 2005 in Australia.

## 1.7. Probability of dying from non-surgical causes among people with OA

Although OA itself is not a life-threatening disease, its presence increases the risk of overall mortality among the patients [16]. In order to adjust for this excess mortality among OA patients, the probability of dying from non-surgical causes was adjusted as follows:

$$NSdeath_{age}^{OA} = 1 - e^{-\left(\frac{Mort_{age}^{popn}}{OAprev_{age}^{2A+} \times RR + (1 - OAprev_{age}^{2A+}) \times 1}\right)}$$

$$Death_{age}^{popn} = NSdeath_{age}^{OA} \times RR$$

where

$NSdeath_{age}^{OA}$ is the probability of dying among people with OA in each age-group from other causes than hip or knee replacement surgeries;

$Death_{age}^{popn}$ is the probability of dying among people without OA in each age-group;

$Mort_{age}^{popn}$ is the background mortality rate of Australian population for each age-group in 2003; and

$RR$ is the relative risk of dying among people with OA compared to without, which was assumed 1.1 in the Burden of Disease study 2003.

## 1.8. Health gains

Once an OA patient undergoes a hip or knee replacement surgery with success (i.e. survived surgery), the person benefits from an improvement in the quality of life and a reduction in the probability of dying. Such health gains were modelled through the differences in DALYs faced by the people between intervention and comparator by using the DW obtained from the Burden of Disease study 2003. The following sections describe how the health gains were modelled in the simulation.

*Reduction of probability of dying*

It was assumed that the probability of dying would be reduced to the level of population without OA when none of the hips or knees fell in OA grades 2S, 3A and 3S.

$$NSdeath_{age}^{OA} \rightarrow Death_{age}^{popn}.$$

However, if one hip or knee was replaced but not the other one, and if the other one was in OA grades 2S, 3A or 3S, then the person was deemed to be subject to the probability of dying of people with OA.

*Improvements in quality of life*

We first derived the adjusted DW for one hip or knee from the original DW used in the Australian Burden of Disease study 2003. This adjustment was made in order to account for the dual nature of joints so that people with bilateral OA have severer disability than those with unilateral OA. We used the Solver function of Microsoft Excel to calculate the adjusted DW so that it satisfied:

$$(1 - DW_u) \times OAnum_{age}^{hip/knee,2A/2S3A/3S} = (1 - DW_a) \times \left(1 - BIprop_{AA/BB/CC}^{hip/knee}\right) \times OAnum_{all\ age}^{hip/knee,2A/2S3A/3S} + (1 - DW_a)^2 \times BIprop_{AA/BB/CC}^{hip/knee} \times OAnum_{all\ age}^{hip/knee,2A/2S3A/3S}$$

where

$DW_u$ is the original DW of OA obtained from Burden of Disease study 2003 (i.e. per person); and

$DW_a$ is the adjusted DW of OA for one hip or knee (i.e. per joint).

The concept of this equation is to redistribute the total amount of disability of OA experienced by the Australian population in 2003 to the estimated number of affected joints rather than to the number of patients. The total DW for two hips or knees is then calculated:

$$DW_a^{both\ hip/knee} = 1 - (1 - DW_a^{right\ hip/knee}) \times (1 - DW_a^{left\ hip/knee}) .$$

Table G provides the values of both DWs.

Table G: Disability weights

| OA severity | Hip | | Knee | |
|---|---|---|---|---|
| | Original DW | Adjusted DW | Original DW | Adjusted DW |
| 2A | 0.01 | 0.00781 | 0.01 | 0.00622 |
| 2S3A | 0.14 | 0.11190 | 0.14 | 0.08989 |
| 3S | 0.42 | 0.35523 | 0.42 | 0.29309 |

We used different approaches to calculate the effect sizes and post-surgical DWs for hip and knee replacements.

*Hip replacement*

To estimate the effect of hip replacement on the DW, we used the regression model from Briggs et al.[17] to estimate the DW for post replacement.

$$Effect_n^{hip,primary/revision} = \frac{1 - EQpost_{age}}{1 - EQpre_{age}}$$

$$DWpost_{age}^{hip,primary/revision} = Effect_n^{hip,primary/revision} \times DW_a$$

where

$Effect_n^{hip,primary/revision}$ is the effect size of hip replacement for primary replacement or revision;

$EQpre/post$ is the calculated EuroQol score of pre/post hip replacement at each age; and

$DWpost_{age}^{hip,primary/revision}$ is the post hip replacement DW for one hip at each age.

The regression coefficients and their correlations are provided in Tables H and I. We used the median value amongst $DWpost_{age}^{hip,primary/revision}$ calculated for ages between 40 and 100 for all ages. The values of effect sizes provided in Table 2 of the main text were obtained by running a Monte Carlo simulation with 5,000 iterations by assuming normal distributions for each regression coefficient in Table H.

Table H: Regression coefficients for EQ-5D index estimation (hip replacement)

| Variable | Pre-surgery | | | Post-surgery | | |
|---|---|---|---|---|---|---|
| | Coefficient | Standard error | P-value | Coefficient | Standard error | P-value |
| Constant | -0.456 | 0.040 | 0.000 | -1.658 | 0.077 | 0.000 |
| Age | -0.001 | 0.001 | 0.372 | 0.001 | 0.002 | 0.692 |
| Male | -0.188 | 0.031 | 0.000 | -0.226 | 0.058 | 0.000 |
| Revision | 0.042 | 0.038 | 0.267 | 0.503 | 0.058 | 0.000 |

Source: Briggs et al.[17]

Table I: Correlation between coefficients (hip replacement)

| Variable | Pre-surgery | | | | Post-surgery | | | |
|---|---|---|---|---|---|---|---|---|
| | Constant | Age | Male | Revision | Constant | Age | Male | Revision |
| Constant | 1 | | | | 1 | | | |
| Age | -0.839 | 1 | | | -0.838 | 1 | | |
| Male | -0.381 | 0.063 | 1 | | -0.351 | 0.043 | 1 | |
| Revision | -0.196 | 0.010 | -0.029 | 1 | -0.332 | 0.032 | 0.033 | 1 |

Source: Briggs et al.[17]

For an example, the pre-surgery score of a 70 years old male patient who undertakes a revision of his hip implant was calculated as:

$-0.456 - 0.001 \times 70 - 0.188 \times 1 + 0.042 \times 1 = -0.672.$

Since the regression model underwent a non-liner transformation g(y) = $ln$(1–y) where y is the EQ-5D score, the estimated value was back transformed to the original EQ-5D score:

$1 - Exp(-0.672) = 0.489.$

It should be noted that this example used the point estimates of regression coefficients (without accounting for the standard errors). When running the simulation model with uncertainty, the values drawn from the assumed normal distribution of each regression coefficient are constrained by the correlation coefficients provided in Table I so that they are correlated with each other.

*Knee replacement*

To estimate the effect of knee replacement on the DW, we used the literature reporting pre and post scores of EQ-5D, HAQ, and SF-36 (converted to a single index by means of TTU method) [18] and estimated the effect size of individual study as:

$$Effect_n^{knee,M/F} = \frac{\left[\left(1 - PYLD_{mean\ age\ n}^{M/F}\right) - Score_n^{post}\right] \div \left(1 - PYLD_{mean\ age\ n}^{M/F}\right)}{\left[\left(1 - PYLD_{mean\ age+1\ n}^{M/F}\right) - Score_n^{pre}\right] \div \left(1 - PYLD_{mean\ age+1\ n}^{M/F}\right)}$$

where

$Effect_n^{knee,M/F}$ is the effect size of knee replacement for male or female derived from the n$^{th}$ literature;

$PYLD_{mean\ age/+1\ n}^{M/F}$ is the prevalent years lived with disability of the Australian males or females obtained from the Burden of Disease study 2003 at the mean age or one year older of the study sample in the n$^{th}$ literature (this was included to account for the age variations of samples between literature); and

$Score_n^{pre/post}$ is the single index reported or converted from EQ-5D, HAQ, and SF-36 of pre/post knee replacement in the n$^{th}$ literature,

and performed a non-parametric bootstrap with 5,000 iterations to obtain the mean values and 95% CI of the effect size (Male: 0.5202, 0.3888–0.6606; Female: 0.5205, 0.3891–0.6580; other values are provided in Table 2 of the main text). The list of literature used for this modelling is provided in Table J.

The DW of post knee replacement was then calculated as:

$$DWpost_{M/F}^{knee,primary} = DW_a^{knee} \times Effect_{M/F}$$

where

$DWpost_{M/F}^{knee,primary}$ is the post primary replacement DW of knee for male or female;

$DW_a^{knee}$ is the pre replacement DW of one knee; and

$Effect_{M/F}$ is the effect size of knee replacement for male or female obtained from bootstrap.

The post surgery DW for knee revision was derived by applying the information of DW for post replacement DW of hip and post revision DW of hip:

$$DWpost_{M/F}^{knee,revision} = DW_a^{knee} - \frac{DW_a^{hip} - DWpost_{M/F}^{hip,revision}}{DW_a^{hip} - DWpost_{M/F}^{hip,primary}} \times (DW_a^{knee} - DWpost_{M/F}^{knee,primary})$$

where

$DWpost_{M/F}^{knee,revision}$ is the post revision replacement DW of knee for male or female;

$DW_a^{hip}$ is the pre replacement DW of one hip; and

$DWpost_{M/F}^{hip,primary/revision}$ is the post primary or revision replacement DW of hip for male or female.

Finally, the DW of a person at each age was calculated as follows:

$$DW_{age}^{person\ hip/knee} = 1 - \left(1 - DW_a^{both\ hip/knee}\right) \times \left(1 - PYLD_{age}\right)$$

where

$DW_{age}^{person\ hip/knee}$ is the DW of a person who originally had hip or knee OA, may or may not have undergone replacement surgeries for each hip or knee, at each age; and

$DW_a^{both\ hip/knee}$ is the DW of both hips or knees of a person who originally had hip or knee OA, may or may not have undergone replacement surgeries for each hip or knee.

Table J: Literature and indexes included for bootstrap (knee replacement)

| Literature | Sample size | Index | Transformed index | |
|---|---|---|---|---|
| | | | Pre-surgery | Post-surgery |
| Brazier et al.[19] | 109 | EQ-5D | 0.45[a] | 0.54[a] |
| | 109 | HAQ | 0.37[a] | 0.43[a] |
| | 109 | SF-36 | 0.39 | 0.50 |
| van Essen GJ et al.[20] | 73 | SF-36 | 0.47 | 0.56 |
| Bennett KJ et al.[21] | 41 | SF-36 | 0.42 | 0.46 |
| Dawson J et al.[22] | 117 | SF-36 | 0.40 | 0.59 |
| Heck DA et al.[23] | 291 | SF-36 | 0.45 | 0.65 |
| Kiebzak GM et al.[24] | 78 | SF-36 | 0.46 | 0.55 |
| Shields RK et al.[25] | 24 | SF-36 | 0.53 | 0.73 |
| Jones CA et al.[26] | 276 | SF-36 | 0.39 | 0.58 |
| Jones CA et al.[27] | 222 | SF-36 | 0.39 | 0.60 |
| | 35 | SF-36 | 0.37 | 0.54 |
| Bachmeier CJ et al.[28] | 108 | SF-36 | 0.43 | 0.63 |
| Bayley KB et al.[29] | 117 | SF-36 | 0.50 | 0.65 |
| Hozack WJ et al.[30] | 149 | SF-36 | 0.50 | 0.65 |
| Kiebzak GM et al.[31] | 235 | SF-36 | 0.45 | 0.60 |

[a] These indexes are original values

## 1.9. Time to revision of hip and knee implants

Once an OA patient has undergone a jip or knee primary replacement, the next issue is on the durability of implants before they need to be revised. Such time to failure of implants was

assumed to be caused either by short-run or long-run causes. We assumed separate Weibull distributions for each cause and derived two cumulative density curves. The time to revision was modelled as the weighted and normalised sum of these two curves by fitting the estimated values to the observed values from literature (see main text for reference) by means of weighted least square. The Solver function of Microsoft Excel was used for the calculation.

$$TTFcomb_{age} = TTFshort_{age} \times WTshort_{age} + TTFlong_{age} \times (1 - WTshort_{age})$$

where $TTFshort/long/comb_{age}$ is the probability density of time to failure of hip or knee implants at short-term, long-term, or both combined, for primary/revision for hip/knee in each age-group, and $WTshort_{age}$ is the weight of short-term cause of failure of hip or knee implants for primary/revision surgeries in each age-group. We defined the "weight" as the probability of a failure being attributed to short-term cause over long-term, and so the weight of long-term cause is calculated as 1 – (weight of short-term cause). These weights were simultaneously estimated by the Solver function so that the combined mixture distribution would best fit to the observed values. Table K provides the sources of data used in modelling the time to failure/revision of hip and knee implants.

Table K: Data used to model the time to failure of joint implants

| | Hip implant | Knee implant |
|---|---|---|
| *Short-term* | | |
| Primary | Australian joint replacement registry 2008[32] | Australian joint replacement registry 2008[32] |
| Revision | ditto | ditto |
| *Long-term* | | |
| Primary | Schulte et al.,[33] Madey et al.,[34] Callaghan et al.,[35] Callaghan et al.[36] | Rand et al.[37] |
| Revision | Schreurs et al.[38] | Rand & Ilstrup.[39] |

The Weibull parameters estimated to model the time to failure/revision are provided in Table L. Alpha represents the scale parameter and Beta the shape parameter.

Table L Weibull parameters for time to failure of hip or knee implants

| Type | Term | Age | Male | | | Female | | |
|---|---|---|---|---|---|---|---|---|
| | | | Alpha | Beta | Weight | Alpha | Beta | Weight |
| ***Hip*** | | | | | | | | |
| Primary | Short | <55 | 0.87 | 45.51 | 0.18 | 0.56 | 152.96 | 0.23 |
| | | 55-64 | 0.57 | 240.21 | 0.27 | 0.99 | 101.56 | 0.64 |
| | | 65-74 | 0.66 | 172.30 | 0.26 | 0.57 | 397.46 | 0.31 |
| | | 75+ | 0.43 | 274.13 | 0.15 | 0.58 | 412.47 | 0.30 |
| | Long | <55 | 2.84 | 51.90 | 0.82 | 2.43 | 38.32 | 0.77 |
| | | 55-64 | 2.38 | 41.67 | 0.73 | 4.25 | 36.90 | 0.36 |
| | | 65-74 | 2.34 | 42.67 | 0.74 | 2.39 | 43.38 | 0.69 |
| | | 75+ | 2.24 | 42.11 | 0.85 | 2.34 | 44.92 | 0.70 |
| Revision | Short | All | 0.39 | 200.36 | 0.70 | 0.39 | 200.36 | 0.70 |
| | Long | All | 8.92 | 26.05 | 0.30 | 8.92 | 26.05 | 0.30 |
| ***Knee*** | | | | | | | | |
| Primary | Short | <55 | 0.55 | 131.27 | 0.50 | 0.66 | 149.67 | 0.68 |
| | | 55-64 | 0.32 | 241.20 | 0.10 | 0.69 | 409.25 | 0.13 |
| | | 65-74 | 0.71 | 846.51 | 0.39 | 0.70 | 947.36 | 0.37 |
| | | 75+ | 1.58 | 658.23 | 0.28 | 1.69 | 559.00 | 0.28 |
| | Long | <55 | 2.98 | 29.88 | 0.50 | 3.64 | 27.20 | 0.32 |
| | | 55-64 | 1.62 | 40.83 | 0.90 | 1.60 | 45.15 | 0.87 |
| | | 65-74 | 1.66 | 28.66 | 0.61 | 1.61 | 34.31 | 0.63 |
| | | 75+ | 1.62 | 31.54 | 0.72 | 1.62 | 32.46 | 0.72 |
| Revision | Short | All | 1.44 | 1.91 | 0.17 | 1.44 | 1.91 | 0.17 |
| | Long | All | 2.48 | 21.13 | 0.83 | 2.48 | 21.13 | 0.83 |

Based on these parameters and the above equation, we derived the probability density curve and cumulative distribution curve to simulate the time to failure of joint implants. Figures A and B provide the cumulative distribution curves, where the red dots represent the observed cumulative implant failure rates from the literature (Table K) and the blue line the estimated values.

Figure A: Cumulative distribution curve of time to revision with observed values (hip implants)

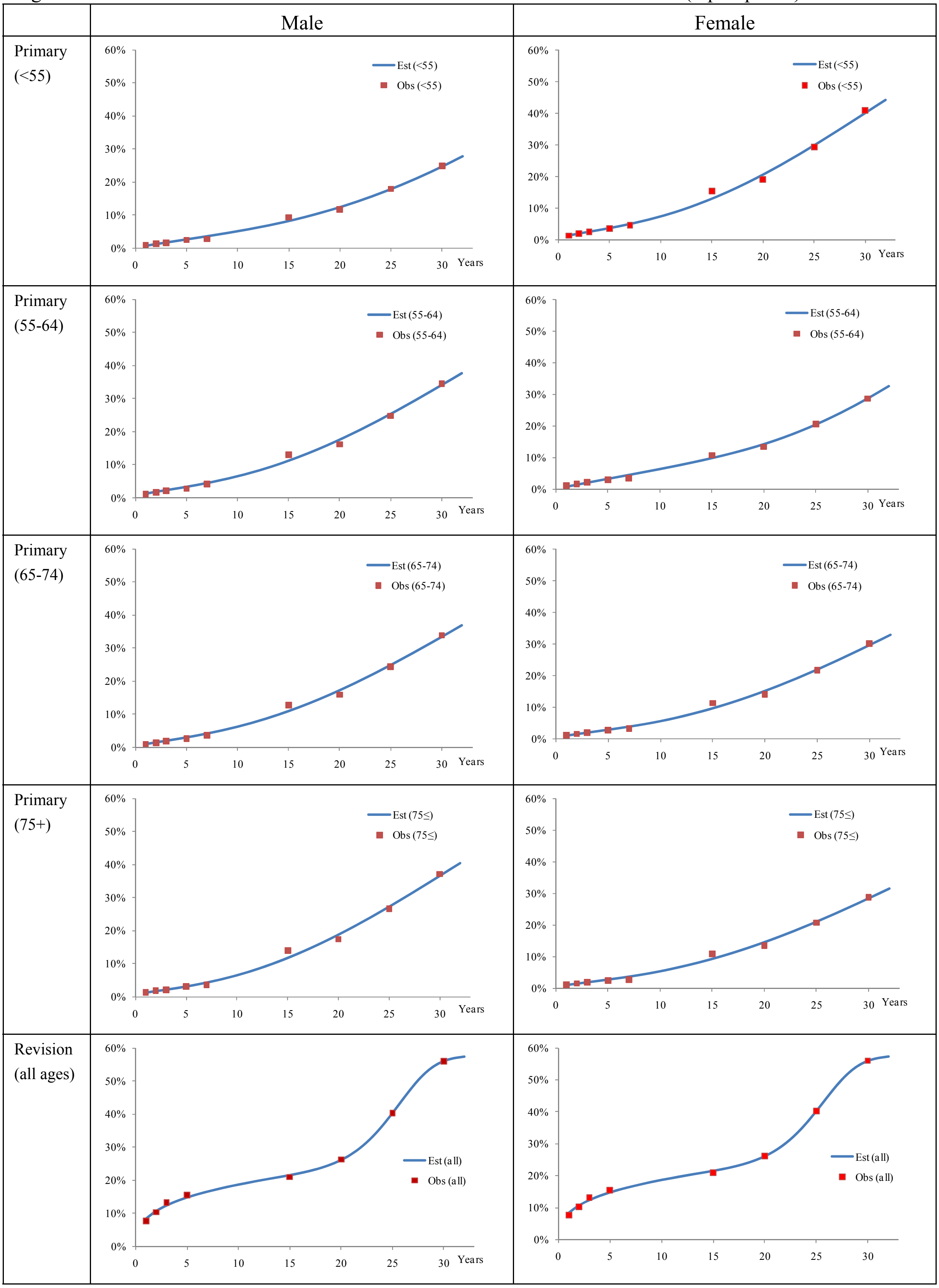


Est: estimated curve; Obs: observed values

Figure B: Cumulative distribution curve of time to revision with observed values (knee implants)

| | Male | Female |
|---|---|---|
| Primary (<55) | 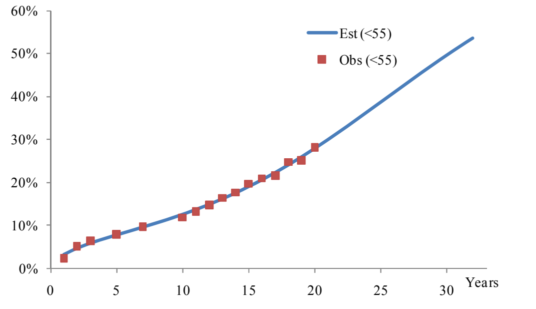 | 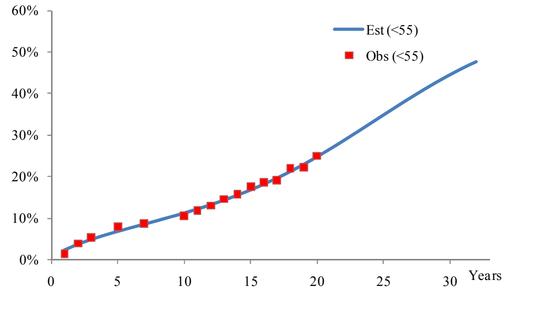 |
| Primary (55-64) | 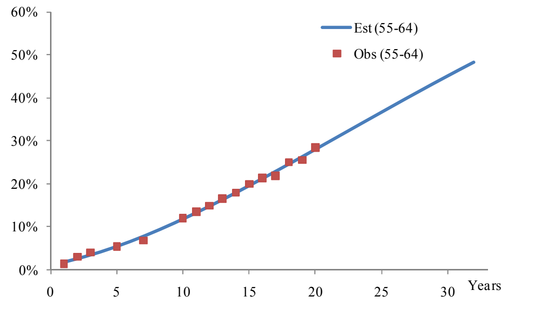 | 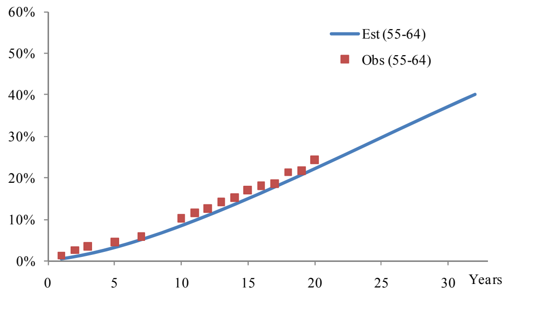 |
| Primary (65-74) | 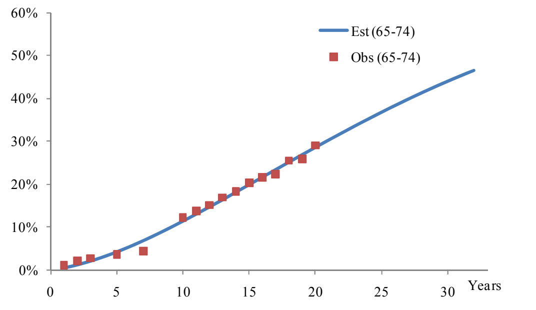 | 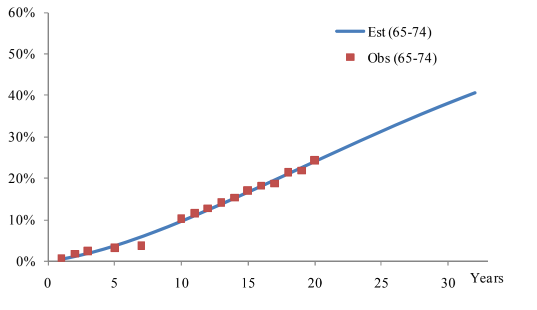 |
| Primary (75+) | 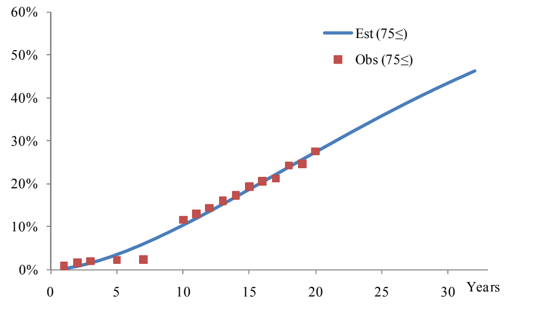 | 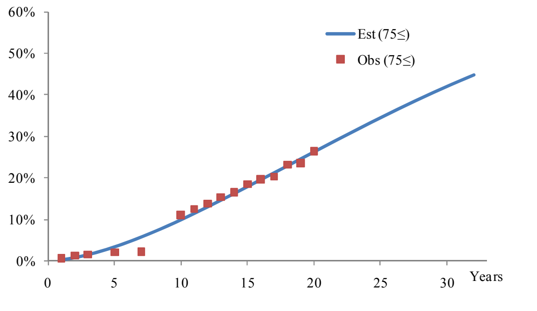 |
| Revision (all ages) | 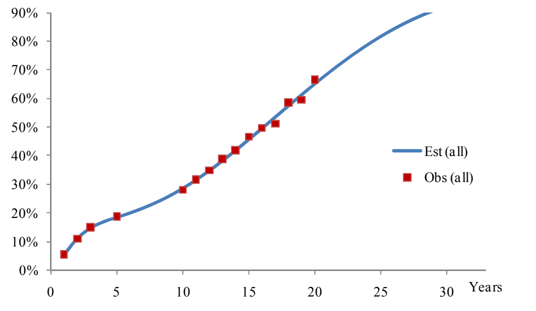 | 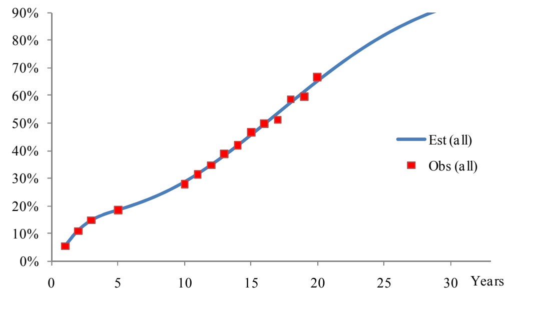 |

Est: estimated curve; Obs: observed values

Similarly, Figure C provides the probability density curve modelled from the estimated parameters.

Figure C: Probability density curve of time to revision

| | Hip implants | Knee implants |
|---|---|---|
| Male | 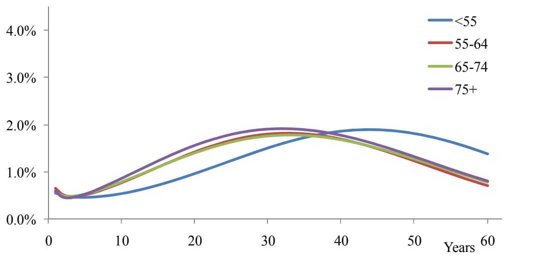 | 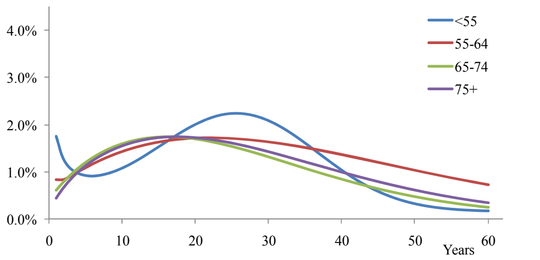 |
| Female | 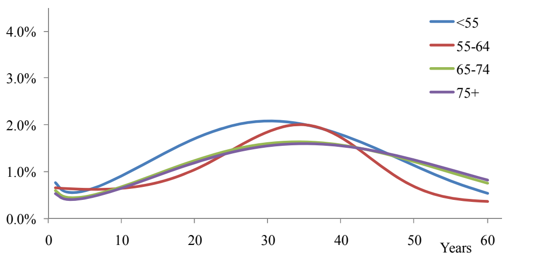 | 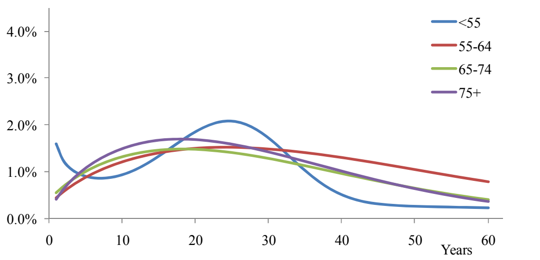 |
| Revision (male & female) | 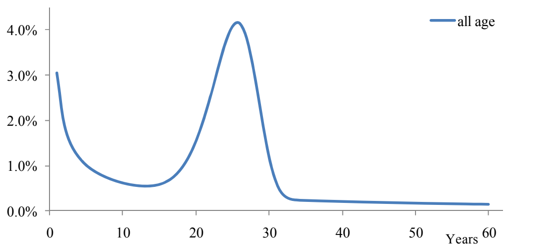 | 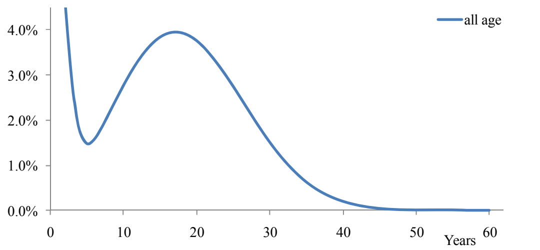 |

## 1.10. Survival curve of implants (primary joint replacement)

Sections 1.1–1.9 provided all details of the mathematical backgrounds and assumptions made to derive the epidemiological parameters. Before proceeding to the estimation of costs, we ran the simulation to derive the survival curves of patients who underwent primary replacement and the primary implants. The results are provided in Figure D.

Figure D: Survival curve for primary implants

| | Hip implants | Knee implants |
|---|---|---|
| Male | 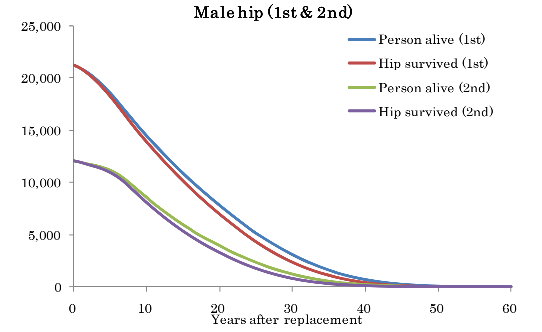 | 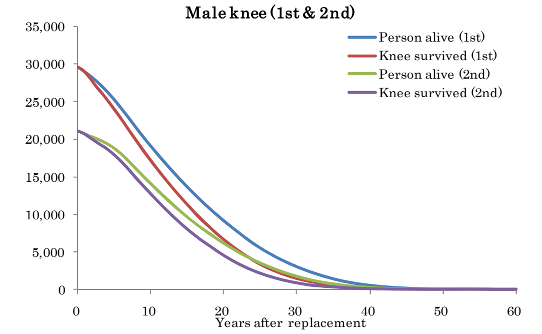 |
| Female | 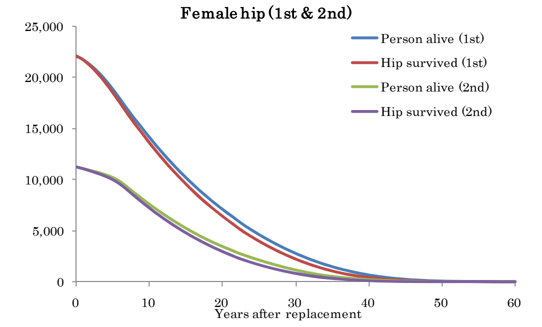 | 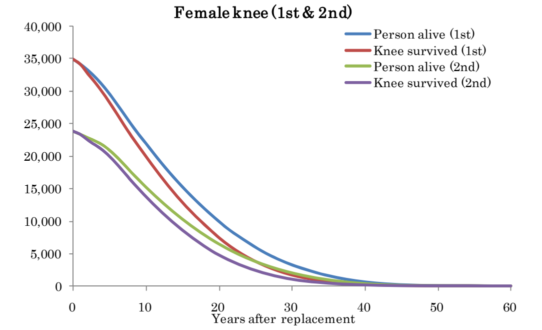 |
| 1st joint | 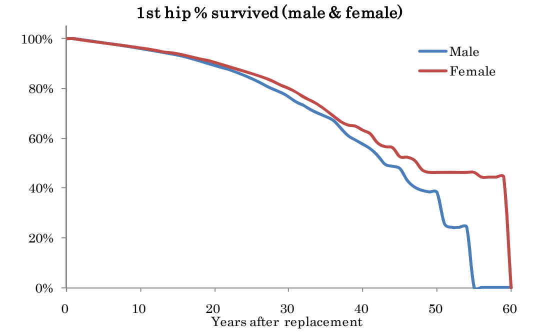 | 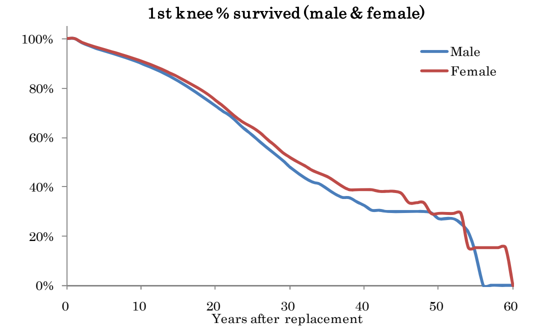 |
| 2nd joint | 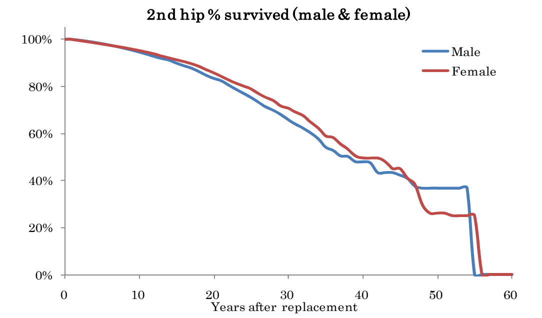 | 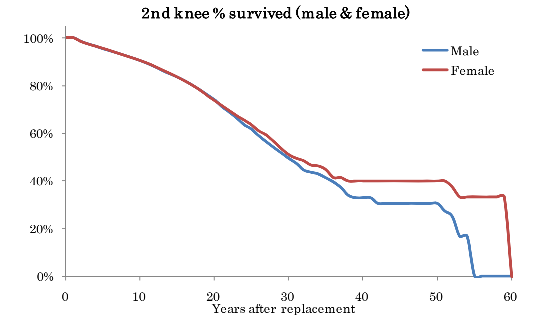 |

Person alive(1st): number of people who are alive X years after primary replacement of the 1st joints

Person alive (2nd): number of people who are alive Y years after primary replacement of the 2nd joints

## 2. Costs

### 2.1. Costs of hip and knee replacement surgeries

The information on costs for hip and knee surgeries were obtained from the Australian Hospital Statistics 2003-04 and the National Hospital Cost Data Collection 2003-04[40,41]. Although the reported costs from these sources were most accurate, they did not provide as detailed information as was necessary for our study. Therefore, we made some assumptions in separating the cost information to be able to be used in the analysis. Table M provides the original cost information for each item that was available from the above sources.

Table M: Costs of hip and knee replacements

| DRG | Item | No. of separations | Average cost per DRG | Average length of stay |
|---|---|---|---|---|
| I03A | Hip Revision + Cscc[a] | 558 | 30,648 | 19.01 |
| I03B | Hip Replac+Cscc/Hip Revsn-Cscc | 5,222 | 16,744 | 12.77 |
| I03C | Hip Replacement - Cscc | 6,687 | 13,648 | 7.52 |
| I04A | Knee Replacemt & Reattach+Cscc | 856 | 19,620 | 14.17 |
| I04B | Knee Replacemt & Reattach-Cscc | 8,318 | 13,640 | 7.08 |

a Catastrophic or severe complications and comorbidities

The issues in using these data were two-fold. First, the probability of having a complication in the surgery had to be estimated for both primary replacement and revisions as input parameters to the model. However, item I03B included both hip replacements with complications and hip revisions without complications that had to be separated to estimate the probability of complications. Second, no information was provided for the number and average costs of knee revisions. Therefore, we made the following assumptions and derived the necessary information.

*Probability of having complications*

First the number of hip replacement + Cscc and hip revision – Cscc were separated assuming that the overall proportion between primary hips and revision hips should be the same as the proportion reported in the Australian National Joint Replacement Registry. The Solver function was used to calculate the number of each case by satisfying the following equation:

$$\frac{NOS_{rev\ \pm Cscc}^{hip}}{NOS_{prim\ \pm Cscc}^{hip}} = \frac{NOS_{rev\ JRR}^{hip}}{NOS_{prim\ JRR}^{hip}}$$

where

$NOS_{prim/rev,+Cscc/-Cscc}^{hip}$ is the number of hip surgeries conducted for primary/revision replacement for Cscc/non-Cscc cases for each DRG code

$NOS_{JRR}^{hip}$ is the number of hip surgeries recorded for 2003 in the Australian National Joint Replacement Registry for primary/revision hip surgeries.

Second, the probabilities of having complications for hip and knee replacements were calculated as:

$$Prob_{prim/rev,+Cscc}^{hip} = \frac{NOS_{prim/rev+Cscc}^{hip}}{NOS_{prim/rev-Cscc}^{hip} + NOS_{prim/rev+Cscc}^{hip}}$$

$$Prob_{prim+Cscc}^{knee} = \frac{NOS_{I04A}}{NOS_{I04A} + NOS_{I04B}}$$

$$Prob_{rev+Cscc}^{knee} = Prob_{prim+Cscc}^{knee} \times \frac{Prob_{rev+Cscc}^{hip}}{Prob_{prim+Cscc}^{hip}}$$

where

$NOS_{I04A/I04B}$ is the number of separations for each DRG code

$Prob_{prim/rev,+Cscc}^{hip/knee}$ is the proportion of Cscc cases for primary /revision hip surgeries for hip or knee.

In estimating the probability of complications for knee revisions, we assumed that the proportional probability difference between + Cscc and – Cscc for hip surgeries applies to knee surgeries as well.

The derived probabilities of complications are provided in Table N.

Table N: Probability of having Cscc

| | Primary | Revision |
|---|---|---|
| Hip | 0.36 | 0.27 |
| Knee | 0.09 | 0.07 |

*Intervention costs for each item*

The costs of hip and knee replacement surgeries were calculated as follows:

$$Cost_{prim-Cscc}^{hip} = CDRG_{I03C}$$
$$Cost_{prim+Cscc}^{hip} = CDRG_{I03B}$$
$$Cost_{rev-Cscc}^{hip} = CDRG_{I03B}$$
$$Cost_{rev+Cscc}^{hip} = CDRG_{I03A}$$
$$Cost_{prim-Cscc}^{knee} = CDRG_{I04B}$$
$$Cost_{prim+Cscc}^{knee} = CDRG_{I04A}$$
$$Cost_{rev-Cscc}^{knee} = CDRG_{I04A}$$
$$Cost_{rev+Cscc}^{knee} = Cost_{rev-Cscc}^{knee} \times \frac{CDRG_{I03A}}{CDRG_{I03B}}$$

where

$Cost_{prim/rev,+Cscc/-Cscc}^{hip,/knee}$ is the cost per primary or revision surgery for hip or knee with or without Cscc

$CDRG_{I03A/I03B/I03C/I04A/I04B}$ is the unit cost for each AR-DRG code

It was assumed that the proportional cost difference between hip revision + Cscc and hip revision – Cscc applies to knee surgeries as well.

## 2.2. Average length of stay

The average length of stay for each surgical pattern was calculated from Table M following the same logic as the costs for hip and knee replacements.

## 2.3. Surgery-related costs

Apart from the costs that are directly associated with hip and knee replacement surgeries, OA patients visit the hospitals pre- and post-surgeries that do not involve admissions (e.g. pre-surgical consultations, pathology tests etc.). Such costs are deemed surgery-related costs, and were estimated from the Disease Costs and Impact Study 2000-01(DCIS 2000-01)[42] as follows:

$$Cost_{surg-related} = \frac{Cost_{2000-01}^{Non-admit} + Cost_{2000-01}^{Pathology}}{No_{2000-01}^{OA\ surgery}} \times \frac{Def_{2003}}{Def_{2001}}$$

where

$Cost_{surg-related}$ is the surgery-related cost to the government other than $Cost_{primary,revision}^{hip,knee}$

$Cost_{2000-01}^{Non-admit,pathology}$ is the costs for hospital non-admitted cases and pathology for males and females from DCIS 2000-01

$Cost_{2000-01}^{OA\ surgery}$ is the number of all types of hip and knee replacements conducted for OA patients in 2000-01 for males and females (obtained from the Joint Replacement Registry)[4]

$Def_{2001,2003}$ is the health price deflator for 2001 and 2003[43].

The assumptions set here included:

- Sum of the costs of hospital non-admitted cases and pathology would well approximate the pre/post surgery-related costs to the government;
- The amount does not differ between, hip and knee, primary and revision surgeries, males and females at all ages;
- DCIS only includes government's cost; and
- There is no sampling error (since the data covers the entire country) and so there is no uncertainty distribution given.

## 2.4. Patient's out-of-pocket costs

Although direct costs associated with hip and knee replacement surgery are deemed to be borne solely by the government, there are other costs associated with pre- and post-surgery that are borne by the patients. This may include co-payment for non-admitted hospital visits, medications, travel costs, etc. Such patient's out-of pocket costs for hip and knee replacements were calculated from March et al.[44] as:

$$Cost_{OOP\ med}^{hip,knee} = Cost_{literature}^{hip,knee} \times \frac{Def_{2003}}{Def_{1995}}$$

$$Cost_{OOP\ fuel}^{hip,knee} = Price_{2003}^{fuel} \times Dist \times 2 \times Visits_{pre\&post}$$

where

$Cost_{OOP\ med}^{hip,knee}$ is the out-of-pocket payment for medical services for a patients undergoing hip or knee replacements surgeries

$Cost_{literature}^{hip,knee}$ is the out of pocket payment of a patient for three months pre/post hip or knee replacements surgeries from the literature

$Cost_{OOP\ fuel}^{hip,knee}$ is the average fuel costs for pre- and post- surgeries medical visits

$Price_{2003}^{fuel}$ is the average fuel price per km in 2003 (assumed to be AUD 0.58per km)

$Dist$ is the average distance between residence and destinations (assumed to be 6.4km)

$Visits_{pre/post}$ is the average number of visits pre- and post surgery from March et al.[44]

The assumptions set here included:

- The out-of-pocket payment of patients during three month prior and post surgery are all related to hip or knee replacement surgeries;
- The amount is the same between primary and revision surgeries, between males and females at all ages; and
- Gamma distribution is assumed for uncertainties.

## 2.5. Patient's time costs for surgery and recuperation

While there is no out-of-pocket costs falling on the patients during hospital admissions and surgeries, there is an opportunity costs associated with the time of patients in the hospital. Suche patient's time costs for surgery and recuperation were estimated from the Average Weekly Earnings 2003[45] and Labour Force Statistics 2003[46] as follows:

$$Earn_{M,F}^{hour} = Earn_{M,F}^{week}$$

$$Leisure_{M,F}^{hour} = Leisure_{M,F}^{week}$$

$$Timecost_{M,F}^{day} = \left( Earn_{M,F}^{hour} \times \frac{Civ_{M,F}^{working}}{Civ_{M,F}^{total}} + Leisure_{M,F}^{hour} \times \frac{Civ_{M,F}^{not\ working}}{Civ_{M,F}^{total}} \right) \times 7.25hrs)$$

$$Timecost_{M,F}^{hip,knee} = Timecost_{M,F}^{day} \times ALOS^{hip.knee/\ primary,revision} \times 2$$

where

$Earn_{M,F}^{hour,day,week}$ is the hourly/daily/weekly earnings of males and females who are working
$Leisure_{M,F}^{hour,day,week}$ is the hourly/daily/weekly time cost of males and females who are not working
$Timecost_{M,F}^{day}$ is the average daily time cost of males and females
$Civ_{M,F}^{working,not\ working,total}$ is the number of male and female civilians who are working/not working/total within the age range of 15-69
$Timecost_{M,F}^{hip/knee}$ is the time cost of males and females associated with hip or knee replacement surgeries and recuperation
$ALOS_{M,F}^{hip/knee,primary/revision}$ is the average length of stay obtained from the Australian Hospital Statistics 2003-04[40] depending on hip or knee and primary or revision surgeries.

The assumptions set here included:

- The time cost for those not working is 25% of those who are working;
- The recuperation time is same as the length of stay;
- The time cost has a Triangular distribution; and
- The length of stay has a Gamma distribution.

## 2.6. Patient's time costs for pre-surgery medical visits

Similarly to the time costs associated with hospital admissions and surgeries, the non-admitted hospital visits also take time off the patients and hence there is an opportunity cost. The patient's time

costs for pre-surgery medical visits were estimated from March et al.[44] as:

$$Timevisits_{M,F}^{hip/knee} = Timecost_{M,F}^{day}/7.25 \times Time \times Vists_{pre}$$

where

$Timevisits_{M,F}^{hip/knee}$ is the time cost of males and females associated with pre-surgery visits for hip and knee replacements

$Time$ is the total time for travel, waiting, and consultation (assumed to be: 0.25 hours for one way travel; 0.5 hours for waiting; and 0.25 hours for consultation).

## 2.7. Cost offsets (non-surgical therapies for OA)

The costs for non-surgical therapies for OA management are deemed to be offset once the person undergoes hip or knee replacement surgery. The costs for OA management were obtained from DCIS 2000-01[42]. First, we estimated the annual health expenditure consumed by a person with OA. Whilst it is possible to simply divide the total annual expenditure on OA by the total number of people with OA to derive the average cost per OA patient, it is unlikely that the people with mild OA and severe OA are consuming the same amount. Therefore, we analysed each cost category (e.g. hospital attendance, pharmaceutical with and without prescription etc.) and assigned each item to the relevant OA grades proportionally to the number of people in each grade. The sum of the health expenditure assigned to each OA grade was divided by the number of people in each grade to derive the grade specific health spending per person per year. These costs were assumed to be offset after successful interventions. The costs for each severity of OA are provided in Table O.

Table O: Annual cost per individuals with OA for conservative therapy (to be offset) (Unit: AUD)

| Age | Male | | | | Female | | | |
|---|---|---|---|---|---|---|---|---|
| | <2A | 2A | 2S3A | 3S | <2A | 2A | 2S3A | 3S |
| 40–44 | 144 | 473 | 911 | 1,785 | 63 | 844 | 2,005 | 4,328 |
| 45–54 | 63 | 389 | 844 | 1,753 | 66 | 1,093 | 2,165 | 4,309 |
| 55–64 | 72 | 497 | 1,013 | 2,045 | 81 | 750 | 1,681 | 3,542 |
| 65–74 | 132 | 489 | 1,040 | 2,141 | 79 | 529 | 1,169 | 2,450 |
| 75–84 | 96 | 322 | 693 | 1,434 | 139 | 372 | 792 | 1,631 |
| 85+ | 40 | 163 | 446 | 1,013 | 74 | 176 | 419 | 904 |

A: asymptomatic; S: symptomatic

The following describe how each cost for different severities of OA was estimated:

$$Cost_{M,F}^{<2A} = \left(Exp_{M,F}^{Unref} + Exp_{M,F}^{Prof}\right) \times \frac{Num_{M,F}^{<2A}}{Num_{M,F}^{OAall}} \div Num_{M,F}^{<2A} \times \frac{Def_{2003}}{Def_{2001}}$$

$$Cost_{M,F}^{2A} = \left[\left(Exp_{M,F}^{Unref} + Exp_{M,F}^{Prof}\right) \times \frac{Num_{M,F}^{2A}}{Num_{M,F}^{OAall}} + \left(Exp_{M,F}^{image} + Exp_{M,F}^{pharmOTC}\right) \times \frac{Num_{M,F}^{2A}}{Num_{M,F}^{2A+}}\right] \div Num_{M,F}^{2A} \times \frac{Def_{2003}}{Def_{2001}}$$

$$Cost_{M,F}^{2S3A} = \Bigg[\left(Exp_{M,F}^{Unref} + Exp_{M,F}^{Prof}\right) \times \frac{Num_{M,F}^{2S3A}}{Num_{M,F}^{OAall}} + \left(Exp_{M,F}^{image} + Exp_{M,F}^{pharmOTC}\right) \times \frac{Num_{M,F}^{2S3A}}{Num_{M,F}^{2A+}} + \left(Exp_{M,F}^{med} + Exp_{M,F}^{pharmPr}\right) \times \frac{Num_{M,F}^{2S3A}}{Num_{M,F}^{2S3A} + Num_{M,F}^{3S} \times DW^{3S}/DW^{2S3A}}\Bigg] \div Num_{M,F}^{2S3A} \times \frac{Def_{2003}}{Def_{2001}}$$

$$Cost_{M,F}^{3S} = \Bigg[\left(Exp_{M,F}^{Unref} + Exp_{M,F}^{Prof}\right) \times \frac{Num_{M,F}^{3S}}{Num_{M,F}^{OAall}} + \left(Exp_{M,F}^{image} + Exp_{M,F}^{pharmOTC}\right) \times \frac{Num_{M,F}^{3S}}{Num_{M,F}^{2A+}} + \left(Exp_{M,F}^{med} + Exp_{M,F}^{pharmPr}\right) \times \frac{Num_{M,F}^{3S}}{Num_{M,F}^{2S3A} + Num_{M,F}^{3S} \times DW^{3S}/DW^{2S3A}} \times \frac{DW^{3S}}{DW^{2S3A}}\Bigg] \div Num_{M,F}^{2S3A} \times \frac{Def_{2003}}{Def_{2001}}$$

where

$Cost_{M,F}^{2A,2S3A,3S}$ is the annual cost for OA management per person for each severity

$Exp_{M,F}^{unref,prof,image,pharmOTC,pharmPr,med}$ is the expenditure for each cost category in 2000-01 (unreferred attendances, other professionals, imaging, pharmaceuticals over the counter, pharmaceuticals requiring prescriptions, and other medical services)

$Num_{M,F}^{<2A,2A,2S\&3A,3S,2A-3S,OAall}$ is the number of population with different OA severity.

These costs were added to the comparator in the analysis to account for the cost that would be saved from hip and knee replacements.

**NB:** There would still be some recurrent costs after replacement surgeries to follow-up hip or knee implants. We treated such costs to be part of non-surgical therapies which would not be offset by the intervention (rather than part of the intervention costs). We assumed that such recurrent costs are equivalent to the costs of non-surgical therapies required for OA grade 2A at three years intervals (this grade was selected since it includes imaging which would be the main service to be provided for follow-up), and deducted from the amount of cost offset. Under this assumption, such recurrent costs were not included in the "without cost offset" scenario.

The assumptions set here included:
- The assumption set above;
- <2A patients only utilise unreferred attendances and other professionals;
- 2A patients utilise <2A costs + imaging and pharmaceuticals over the counter;
- 2S & 3-4A and 3-4S patients utilise 2A costs + pharmaceuticals requiring prescriptions and other medical services which are weighted by the proportion of DWs of 2S&3-4A and 3-4S;
- Costs for hospital admissions are not used for non-surgical therapies;
- Costs for aged care homes and research are not offset by the intervention; and
- There is no sampling error (since the data covers the entire country) and so there is no uncertainty distribution given.

## 3. Additional results

### 3.1. Sex-specific results

Table P provides the sex-specific cost-effectiveness ratios for hip and knee replacements.

Table P: Incremental cost-effectiveness ratio (sex-specific)

| Scenario | Hip (AUD/DALY averted) | | Knee (AUD/DALY averted) | |
|---|---|---|---|---|
| | Mean | 95%UI | Mean | 95%UI |
| *With cost offset* | | | | |
| Without time cost (M) | 4,100 | 3,600−4,900 | 11,000 | 8,500−14,000 |
| Without time cost (F) | 3,100 | 2,700−3,600 | 9,000 | 7,400−12,000 |
| With time costs (M) | 5,800 | 4,800−7,100 | 14,000 | 10,000−18,000 |
| With time costs (F) | 4,300 | 3,600−5,300 | 12,000 | 8,900−15,000 |
| *Without cost offset* | | | | |
| Without time cost (M) | 9,900 | 8,700−12,000 | 19,000 | 15,000−25,000 |
| Without time cost (F) | 10,000 | 9,200−12,000 | 19,000 | 15,000−25,000 |
| With time costs (M) | 12,000 | 10,000−14,000 | 22,000 | 17,000−28,000 |
| With time costs (F) | 11,000 | 10,000−13,000 | 21,000 | 16.000−28,000 |

NB: The values are rounded to two digits of significance

### 3.2. Age-group specific results

Table Q provides the age-group specific results of the analysis (uncertainties are not included).

Table Q: Incremental cost-effectiveness ratio (age-group specific)

| Scenario | Hip (AUD/DALY averted) | | Knee (AUD/DALY averted) | |
|---|---|---|---|---|
| | Without time cost | With time cost | Without time cost | With time cost |
| *With cost offset* | | | | |
| 40-49 | Dominant* | 770 | 2,900 | 5,000 |
| 50-59 | 1,000 | 2,200 | 4,200 | 6,200 |
| 60-69 | 3,300 | 4,700 | 8,800 | 11,000 |
| 70-79 | 8,700 | 11,000 | 16,000 | 19,000 |
| 80-90 | 17,000 | 19,000 | 25,000 | 29,000 |
| 90+ | 32,000 | 37,000 | 47,000 | 53,000 |
| *Without cost offset* | | | | |
| 40-49 | 7,100 | 8,200 | 14,000 | 16,000 |
| 50-59 | 8,200 | 9,500 | 15,000 | 17,000 |
| 60-69 | 9,700 | 11,000 | 18,000 | 20,000 |
| 70-79 | 14,000 | 16,000 | 22,000 | 25,000 |
| 80-90 | 21,000 | 23,000 | 30,000 | 34,000 |
| 90+ | 36,000 | 41,000 | 52,000 | 58,000 |

* Dominant refers to health gain with net-cost saving.

### 3.3. Results from sensitivity analyses

Table R provides the results by assuming an extreme correlation between the left and right joints disease progression.

Table R Incremental cost-effectiveness ratio

| Scenario | Original (AUD/DALY averted)* | | **±0.99 correlation** (AUD/DALY averted) | |
|---|---|---|---|---|
| | Hip | Knee | Hip | Knee |
| *With cost offset* | | | | |
| Without time cost | 3,600 | 10,000 | 3,700 | 10,000 |
| With time costs | 5,100 | 12,000 | 5,100 | 13,000 |
| *Without cost offset* | | | | |
| Without time cost | 10,000 | 19,000 | 10,000 | 19,000 |
| With time costs | 12,000 | 21,000 | 12,000 | 21,000 |

* In mean values.

Table S provides the results by assuming that only one joint is affected by OA through out the life.

Table S: Incremental cost-effectiveness ratio

| Scenario | Original (AUD/DALY averted)* | | **One joint** (AUD/DALY averted) | |
|---|---|---|---|---|
| | Hip | Knee | Hip | Knee |
| *With cost offset* | | | | |
| Without time cost | 3,600 | 10,000 | Dominant** | 1,700 |
| With time costs | 5,100 | 12,000 | 800 | 3,000 |
| *Without cost offset* | | | | |
| Without time cost | 10,000 | 19,000 | 6,900 | 11,000 |
| With time costs | 12,000 | 21,000 | 7,900 | 12,000 |

* In mean values.

** Dominants refers to more health gains at lower cost.

Table T provides the results of hip replacement by using EQ-5D as the outcome measure.

Table T: Incremental cost-effectiveness ratio (hip)

| Scenario | Original (AUD/DALY averted)* | | **EQ-5D** (AUD/QALY averted) | |
|---|---|---|---|---|
| | Hip (2 joints) | Hip (1 joint) | Hip (2 joints) | Hip (1 joint) |
| *With cost offset* | | | | |
| Without time cost | 3,600 | Dominant** | 3,900 | Dominant** |
| With time costs | 5,100 | 800 | 5,400 | 780 |
| *Without cost offset* | | | | |
| Without time cost | 10,000 | 6,900 | 11,000 | 6,500 |
| With time costs | 12,000 | 7,900 | 12,000 | 7,400 |

* In mean values.

** Dominants refers to more health gains at lower cost.

### 3.4. Testing for internal consistency

In order to test the internal consistency of the model, the model was verified by comparing the proportions of total joint replacements occurring in each sex/age-group for a given year between the joint registry and our simulation. Table U provides the comparisons.

Table U: Comparison of observed vs. simulated proportions of sex/age-specific joint replacements per year

| Group | Hip | | Knee | |
|---|---|---|---|---|
| | Observed | Simulated | Observed | Simulated |
| Male <55 | 5% | 9% | 3% | 4% |
| Male 55–64 | 10% | 11% | 10% | 11% |
| Male 65–74 | 15% | 14% | 16% | 15% |
| Male75–84 | 12% | 10% | 13% | 11% |
| Male 85+ | 2% | 2% | 1% | 2% |
| Female <55 | 7% | 7% | 4% | 4% |
| Female 55–64 | 12% | 11% | 13% | 12% |
| Female 65–74 | 20% | 17% | 22% | 20% |
| Female75–84 | 15% | 16% | 17% | 18% |
| Female 85+ | 3% | 5% | 2% | 4% |
| Total | 100% | 100% | 100% | 100% |

## Reference


1. Begg S, Vos T, Barker B, Stevenson C, Stanley L, et al. (2007) The burden of disease and injury in Australia 2003. Canberra: Australian Institute of Health and Welfare.
2. Felson D (1990) The epidemiology of knee osteoarthritis: results from the Framingham Osteoarthritis Study. Seminars in arthritis and rheumatism 20: 42-50.
3. Guccione A, Felson D, Anderson J (1990) Defining arthritis and measuring functional status in elders: methodological issues in the study of disease and physical disability. Am Public Health Assoc. pp. 945-949.
4. Australian Orthopaedic Association National Joint Replacement Registry (2004) Annual report. Adelaide: Australian Orthopaedic Association.
5. Dawson J, Linsell L, Zondervan K, Rose P, Randall T, et al. (2004) Epidemiology of hip and knee pain and its impact on overall health status in older adults. Rheumatology 43: 497-504.
6. Tepper S, Hochberg MC (1993) Factors associated with hip osteoarthritis: data from the first national health and nutrition examination survey (NHANES-1). American Journal of Epidemiology 137: 1081-1088.
7. Hochberg M, Lethbridge-Cejku M, Scott W, Reichle R, Plato C, et al. (1995) The association of body weight, body fatness and body fat distribution with osteoarthritis of the knee: data from the Baltimore Longitudinal Study of Aging. Journal of Rheumatology 22: 488-493.
8. Mannoni A, Briganti MP, Di Bari M, Ferrucci L, Costanzo S, et al. (2003) Epidemiological profile of symptomatic osteoarthritis in older adults: a population based study in Dicomano, Italy. Annals of the Rheumatic Diseases 62: 576-578.
9. Ingvarsson T, Hagglund G, Lohmander LS (1999) Prevalence of hip osteoarthritis in Iceland. Annals of the Rheumatic Diseases 58: 201-207.
10. Davis MA, Ettinger WH, Neuhaus JM (1990) Obesity and osteoarthritis of the knee: evidence from the national health and nutrition examination survey (NHANES I). Seminars in Arthritis and Rheumatism 20: 34-41.
11. Zhang Y, Xu L, Nevitt MC, Aliabadi P, Yu W, et al. (2001) Comparison of the prevalence of knee osteoarthritis between the elderly Chinese population in Beijing and whites in the United States: The Beijing osteoarthritis study. Arthritis & Rheumatism 44: 2065-2071.
12. Ledingham J, Regan M, Jones A, Doherty M (1995) Factors affecting radiographic progression of knee osteoarthritis. Annals of the Rheumatic Diseases 54: 53-58.
13. Lacey R, Thomas E, Duncan R, Peat G (2008) Gender difference in symptomatic radiographic knee osteoarthritis in the Knee Clinical Assessment - CAS(K): a prospective study in the general population. BMC Musculoskeletal Disorders 9: 82.
14. Dieppe P, Cushnaghan J, Tucker M, Browning S, Shepstone L (2000) The Bristol 'OA500 study': progression and impact of the disease after 8 years. Osteoarthritis & Cartilage 8: 63-68.
15. Canadian Institute for Health Information (2007) Canadian joint replacement registry report: total hip and total knee replacements in Canada. Ottawa: Canadian Institute for Health Information.
16. Nüesch E, Dieppe P, Reichenbach S, Williams S, Iff S, et al. (2011) All cause and disease specific mortality in patients with knee or hip osteoarthritis: population based cohort study. BMJ: British Medical Journal 342.
17. Briggs A, Sculpher M, Dawson J, Fitzgerald R, Murray D, et al. (2003) Modelling the cost-effectiveness of primary hip replacement: how cost-effective is the Spectron compared to the Charnley prosthesis? York: Centre for Health Economics, The University of York.
18. Segal L, Day S, Chapman A, Osborne R (2004) Priority setting in osteoarthritis. Melbourne: Centre for Health Economics, Monash University.
19. Brazier JE, Harper R, Munro J, Walters SJ, Snaith ML (1999) Generic and condition-specific outcome measures for people with osteoarthritis of the knee. Rheumatology 38: 870-877.
20. Van Essen GJ, Chipchase LS, O'Connor D, Krishnan J (1998) Primary total knee replacement: short-term outcomes in an Australian population. Journal of Quality in Clinical Practice 18: 135-142.
21. Bennett K, Torrance G, Moran L, Smith F, Goldsmith C (1997) Health state utilities in knee replacement surgery: the development and evaluation of McKnee. Journal of Rheumatology

24: 1796-1805.
22. Dawson J, Fitzpatrick R, Murray D, Carr A (1998) Questionnaire on the perceptions of patients about total knee replacement. Journal of Bone & Joint Surgery - British Volume 80: 63-69.
23. Heck D, Robinson R, Partridge C, Lubitz R, Freund D (1998) Patient outcomes after knee replacement. Clinical Orthopaedics and Related Research 356: 93-110.
24. Kiebzak G, Vain P, Gregory A, Mokris J, Mauerhan D (1997) SF-36 general health status survey to determine patient satisfaction at short-term follow-up after total hip and knee arthroplasty. Journal of the Southern Orthopaedic Association 6: 169-172.
25. Shields R, Enloe L, Leo K (1999) Health related quality of life in patients with total hip or knee replacement. Archives of Physical Medicine and Rehabilitation 80: 572-579.
26. Jones C, Voaklander D, Johnston D, Suarez-Almazor M (2000) Health related quality of life outcomes after total hip and knee arthroplasties in a community based population. Journal of Rheumatology 27: 1745-1752.
27. Jones C, Voaklander D, Johnston D, Suarez-Almazor M (2001) The effect of age on pain, function, and quality of life after total hip and knee arthroplasty. Archives of Internal Medicine 161: 454-460.
28. Bachmeier CJ, March LM, Cross MJ, Lapsley HM, Tribe KL, et al. (2001) A comparison of outcomes in osteoarthritis patients undergoing total hip and knee replacement surgery. Osteoarthritis & Cartilage 9: 137-146.
29. Bayley K, London M, Grunkemeier G, Lansky D (1995) Measuring the success of treatment in patient terms. Medical Care.
30. Hozack W, Rothman R, Albert T, Balderston R, Eng K (1997) Relationship of total hip arthroplasty outcomes to other orthopaedic procedures. Clinical Orthopaedics & Related Research 344: 88-93.
31. Kiebzak G, Campbell M, Mauerhan D (2002) The SF-36 general health status survey documents the burden of osteoarthritis and the benefits of total joint arthroplasty: but why should we use it. American Journal of Managed Care 8: 463-474.
32. Australian Orthopaedic Association National Joint Replacement Registry (2008) Annual report. Adelaide: Australian Orthopaedic Association.
33. Schulte KR, Callaghan JJ, Kelley SS, Johnston RC (1993) The outcome of Charnley total hip arthroplasty with cement after a minimum twenty-year follow-up. The results of one surgeon. Journal of Bone & Joint Surgery - American Volume 75: 961-975.
34. Madey SM, Callaghan JJ, Olejniczak JP, Goetz DD, Johnston RC (1997) Charnley total hip arthroplasty with use of improved techniques of cementing. The results after a minimum of fifteen years of follow-up. Journal of Bone & Joint Surgery - American Volume 79: 53-64.
35. Callaghan JJ, Albright JC, Goetz DD, Olejniczak JP, Johnston RC (2000) Charnley total hip arthroplasty with cement: minimum twenty-five-year follow-up. Journal of Bone & Joint Surgery - American Volume 82: 487-497.
36. Callaghan JJ, Templeton JE, Liu SS, Pedersen DR, Goetz DD, et al. (2004) Results of Charnley total hip arthroplasty at a minimum of thirty years. A concise follow-up of a previous report. Journal of Bone & Joint Surgery - American Volume 86: 690-695.
37. Rand JA, Trousdale RT, Ilstrup DM, Harmsen WS (2003) Factors affecting the durability of primary total knee prostheses. Journal of Bone & Joint Surgery - American Volume 85-A: 259-265.
38. Schreurs BW, Bolder SBT, Gardeniers JWM, Verdonschot N, Slooff TJJH, et al. (2004) Acetabular revision with impacted morsellised cancellous bone grafting and a cemented cup: A 15- to 20-year follow-up. Journal of Bone & Joint Surgery - British Volume 86-B: 492-497.
39. Rand J, Ilstrup D (1991) Survivorship analysis of total knee arthroplasty. Cumulative rates of survival of 9200 total knee arthroplasties. Journal of Bone & Joint Surgery - American Volume 73: 397-409.
40. Australian Institute of Health and Welfare (2005) Australian hospital statistics 2003-04. Canberra: Australian Institute of Health and Welfare.
41. Australian Government Department of Health and Ageing (2005) National hospital cost data collection: cost report round 08 (2003-2004) AR-DRGv4.2. Canberra: Australian Government Department of Health and Ageing.

42. Australian Institute of Health and Welfare (2001) Disease costs and impact study 2000-01. Canberra: Australian Institute of Health and Welfare.
43. Australian Institute of Health and Welfare (2005) Health expenditure Australia 2003-04. Canberra: Australian Institute of Health and Welfare.
44. March L, Cross M, Tribe K, Lapsley H, Courtenay B, et al. (2002) Cost of joint replacement surgery for osteoarthritis: the patients' perspective. Journal of Rheumatology 29: 1006-1014.
45. Australian Bureau of Statistics (2004) Average weekly earnings: November 2003. Camberra: Australian Bureau of Ststistics.
46. Australian Bureau of Statistics (2005) Persons not in the labour force. Camberra: Australian Bureau of Ststistics.
